# Supplementary material for: AP2/ERF Transcription Factor NbERF-IX-33 Is Involved in the Regulation of Phytoalexin Production for the Resistance of Nicotiana benthamiana to Phytophthora infestans
Source: Front Plant Sci. 2022 Jan 27;12:821574. doi: 10.3389/fpls.2021.821574 (PMC8830488; doi:10.3389/fpls.2021.821574)
Supplement: Supplementary file 1 [file Data_Sheet_1.PDF]

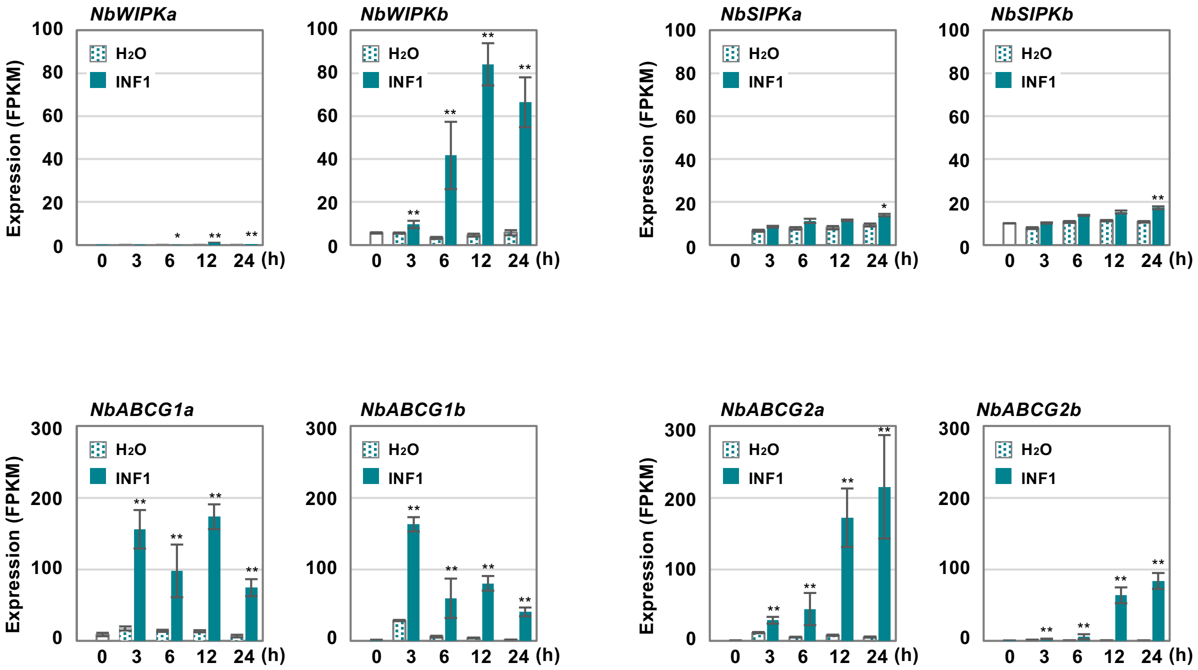

SUPPLEMENTARY FIGURE 1 | Expression profiles of *Nicotiana benthamiana* genes encoding defense-related MAP kinases, *NbWIPK* and *NbSIPK,* and ABC transporters for capsidiol secretion, *NbABCG1* and *NbABCG2*. The gene expression (FPKM value) was determined by RNA-seq analysis of *N. benthamiana* leaves treated with water and 150 nM INF1 for 0 h, 3 h, 6 h, 12 h, 24 h. Data are means ± SE (n = 3). Data marked with asterisks are significantly different from control as assessed by the two-tailed Student's *t*-test: $**P < 0.01$, $*P < 0.05$.

P_*NbEAS1*

-350 ATTTAACTGCTACTTTAAATAATACTATCCACTTAACCAAAAAATGAAAA

ATAAAGTACATAAACTTTAAATAATAGGGAAATTTGGATCAACAGACCCC

-250 AGACGCCAAGAATGAATTAATAGGCTGCTGGTTGGCTGACTAGCTAGTTA

GTGTAAAGTCAAGTAAGGCAACTGGGAAATGATTAGTTGTTTAAATAATT

-150 GGCTGCACTTTTCTCACAACTATATATATATATATATACACTTGTCCCTT

CTCTTCCATTCAAATCATCAGCAATTCAGAGTTCCTAATTTCCTTCTTCC

-50 TTAAAACGAACAAAAACAATACCCTCATCTTTTAATTTATTAGCAATATA

1 ATG

P_*NbEAS2*

-350 ATTTAACTGCTACTTTAAATAATACTATCCACTTAACCAAAAAATGAAAA

ATAAAGTACATAAACTTTAAATAATAGGGAAATTTGGATCAACAGACCCC

-250 AGACGCCAAGAATGAATTAATAGGCTGCTGGTTGGCTGACTAGCTAGTTA

GTGTAAAGTCAAGTAAGGCAACTGGGAAATGATTAGTTGTTTAAATAATT

-150 GGCTGCACTTTTCTCACAACTATATATATATATATATACACTTGTCCCTT

CTCTTCCATTCAAATCATCAGCAATTCAGAGTTCCTAATTTCCTTCTTCC

-50 TTAAAACGAACAAAAACAATACCCTCATCTTTTAATTTATTAGCAATATA

1 ATG

P_*NbEAS3*

-350 TAACTAAGGTGGCTAGACATTTATAATAAAGAGTAGTTAGTGTAAACATT

ATTTTAGAGACGTAGGATGTCCCACAACACAAAATTTTTTATGTAAGAAA

-250 TACTGCTCCTATTATACTGGATCAATTGAGACGCCAAGAAAGAAATCAAA

AGACGCCAAGGAAGAAATATTGTATCAGTAGACTACAGTCAAGTAAGGCA

-150 ACTGAAATGAAGAAATAAAAAAACACTATAAATACTTATGCCTTCTCTTC

CATTTGGGTCATCAGTCTACTTTCTTTTTCTTCCTCGGAGAATTAAGAAG

-50 CCAAAAATTCTCCTATCATTTGTGGTACTAAGAGTATAAATTCTATAGCA

1 ATG

P_*NbEAS4*

-350 TAACTAAGGTGGCTAGACATTTATAATAAAGAGTAGTTAGTGTAAACATT

ATTTTAGAGACGTAGGATGTCCCACAACACAAAATTTTTTATGTAAGAAA

-250 TACTGCTCCTATTATACTGGATCAATTGAGACGCCAAGAAAGAAATCAAA

AGACGCCAAGGAAGAAATATTGTATCAGTAGACTACAGTCAAGTAAGGCA

-150 ACTGAAATGAAGAAATAAAAAAACACTATAAATACTTATGCCTTCTCTTC

CATTTGGGTCATCAGTCTACTTTCTTTTTCTTCCTCGGAGAATTAAGAAG

-50 CCAAAAATTCTCCTATCATTTGTGGTACTAAGAGTATAAATTCTATAGCA

1 ATG

P_*NbEAS5*

-350 CAATTAAATCTCGTCAATTTATTTTTAACCAATCCAAATAAGAAGTTGGA

GAAAGAAAAGACTAAGAAATAGAAGGGAGTTGAATACTCTTAACTAAAGA

-250 GTAGTTACTAGGAAAGTAAACATTGACACAATAAAAAATATCCGTAAGAA

ATACTACTACAATTATATTGGATTCACTGAGACGCCAATAAAGAAATCAA

-150 AAGACGCTGCTGATGATTGGTATATATGGTCAAGTAAGGCAACTGAAATG

AAGAAATAAAAAAGTATTATAAATACTTATGCCTTCTCTACCAATTGTGT

-50 CATCACTCAGAGAATTAATAACACAAAATTCTCCTATAACTTTTATAGCA

1 ATG

P_*NbEAS6*

```
-350 TCTTAATTAAGGCGGCTAGAAATTTAAAGTTTATAATAAAGACCAGTTAC
     TACTATATATTATTTTCCCACGACACTAATTTTTTTCCGTAAGAAATAA
-250 TACTACTATTCAATGAGACACCAATAAAGAAATCAAAAAGCTGGTGATGA
     GTGGTATATATGGTCAAGTAAGGCAACTGAAATGAAGAAATAAAAAAGCA
-150 TTATAAATACTTATGCCTTCTCTACCAATTGTGTCATCACTCAGAGAATT
     AATAACACAAAATTCTCCTATAACTTCTATAGCAATGGCCGCAGCAGCAG
 -50 TTGGCANCAGAGAATTAATAACACAAAATTCTCCTATGACTTTTATAGCA
   1 ATG
```

P_*NbEAS7*

```
-350 TCAATTAATTTCCAACGGAGAGACTAAGAAATGGAAGAGAGTTGAATACT
     CTTAATTAAGGCGGCTAGATATTTAAAATTTATAATAAAGACCAGTTACT
-250 ACTATATATTATTTGCCCACGACACAAAATTTTTTTGCGTAAGAAATAAT
     ACTACTATTCAATGAGACGCCAATAAAGAAATCAAAAAGCTGGTGATGAG
-150 TGGTATATATGGTCAAGTAAGGCAACTGAAATGAAGAAATAGAAAAGCAT
     TATAAATACTTATGCCTTCTCTACCAATTGTGTCATCACTCCACTTTCTT
 -50 TCTTCCCCAGAGAATTAATAACACAAAATTCTCCTATGACTTTTATAGCA
   1 ATG
```

P_*NbEAS8*

```
-350 CGTTTATAATAAAGAGTTGTTACTGGGAAAGTAAACATTATTGTATGATG
     TCCCACAACACAATTTATTTTTAAGTAAGAAATACTACTCGTATTATATT
-250 GGATCAATTGAGACGCCAAGAAAGAAATCAAAAGACGCCAATGAAGAAAT
     ATTGGAACAGTAGACTATATGATATATAGTCAAGTAAGGCAACTGAAATG
-150 AAGAAATAAAAAAATCACTATAAATACCTATGCGTTCTCTTCCGTTTGGG
     TCATCTCACTCATCAGTCTACTTTCTTTCTTCCTCGGAGAATTAAGAACC
 -50 AAAAAATTCTCTTATCATTTGTGGTACTAAGAGTATAACTTTTATTAGCA
   1 ATG
```

P_*NbEAS9*

```
-350 GCAATTTGGCTATTTAAATAATATTATTCAGTTTACGAAAAAAGAATAAA
     GTAATAGACTTTAAATAATTGAGAAACTGGATGAATAGACCACAGACGCC
-250 AACAATGAATCAAAAGGCTGCTGCCTAATGTAAAGTCAAGTAAGACAACT
     GGGAATTTGATTAGGTGATTTTGATCAGTTAAAATATCAACGTGACGTAG
-150 TTGTTTTAAATAATAATTACATGAACAAAAATTAGAGAATTGGCTTTGGG
     TCTCTCACCACTATATATACTTGTCCCTTGCCTTCCATTTAAGTCAACAA
 -50 CTGAGAGTTCCTTAAAATAACAAAAACAATACTCTCATCTTTTATTAGCA
   1 ATG
```

P_*NbEAS10*

```
-350 --------------------------------------------------
     --------------------------------------------------
-250 --------------CGCCCCGTCGCCGACTTCTCCCCTAGTCTTTGGGGT
     GATCAGTTCCTTTCATTCTCCATTGACAATCAGGTAATTTATACTTTCTT
-150 CGTGCAAGCATGCATGGTTTATTTCTTCTTTGTATTTATTGAAAGACATT
     AATTTTGCTAACTTCACTTTTCTTTTTTTAAATACGTTATAAATAGGTTG
 -50 CAGAAACGTATGCTAAAGAGATTGAAGCACTGAAGGAGCAAACGAGGAAT
   1 ATG
```

P_*NbEAH1*

-350 AAGTATTTAGTGAGGAGTTAGTATCAAATTTGTAGCCGCCAAATATGAAA
GTCTTTGTGGGCTATACTTTCGGATTATATAGAAAAAAGAATATATTGCT
-250 TCACAATAATCAGTCCACAATACTAATAATTAATTACATCATTATTGCTC
TCTAGTCAGCTGAATATGAATAATTAATGGTCAACAGGAAAGTAAACAAA
-150 GTCTGCTTCAATGAGATGTTGATCAACAAAAGTTGCAGAAAATATTAGTA
TATTTATCTTTCCATTTCTTGCCAAAACAAATGCCTATAAAAGACCATAC
-50 GCATCCAGACTTTGAAGAATCATCGAATAATCCTCCATTTATCTCCGAAA
1 ATG

P_*NbEAH2*

-350 TTTTATCCATATTTTACCCATTTTTAAAAATTTAGTCGTCCAACCCATTT
TTGGTGAATTATATTAATGGATAATTATTTTTTTAAATCCATTTCGCCGC
-250 CGCTACTCCGAGATTATTAGAGAATTTCCCCCGCATTGCTGTTCTTTGAG
TCAGCTGAGTTGTTTTGACTCTGAAGGAAGTTGAACAGTTAAGTAAACAA
-150 AGTCTGCTTTGACAAACAAATGTTGCAGAAAAATACTACTCTTTCTTTGC
ATATCCCATTCTGGCCAAAACAAAAGCCTATAAAAGACCACACACAACTG
-50 ACCTTTGAATTATATCATCGAACCAAATACTCCTCCATTTATCTCCCAAA
1 ATG

P_*NbEAH3*

-350 AAGTATTTAGTGAGGAGTTAGTATCAAATTTGTAGCCGCCAAATATGAAA
GTCTTTGTGGGCTATACTTTCGGATTATATAGAAAAAAGAATATATTGCT
-250 TCACAATAATCAGTCCACAATACTAATAATTAATTACATCATTATTGCTC
TCTAGTCAGCTGAATATGAATAATTAATGGTCAACAGGAAAGTAAACAAA
-150 GTCTGCTTCAATGAGATGTTGATCAACAAAAGTTGCAGAAAATATTAGTA
TATTTATCTTTCCATTTCTTGCCAAAACAAATGCCTATAAAAGACCATAC
-50 GCATCCAGACTTTGAAGAATCATCGAATAATCCTCCATTTATCTCCGAAA
1 ATG

P_*NbEAH4* (Niben101Scf00072g05004.1)

-350 AAGTATTTAGTGAGGAGTTAGTATCAAATTTGTAGCCGCCAAATATGAAA
GTCTTTGTGGGCTATACTTTCGGATTATATAGAAAAAAGAATATATTGCT
-250 TCACAATAATCAGTCCACAATACTAATAATTAATTACATCATTATTGCTC
TCTAGTCAGCTGAATATGAATAATTAATGGTCAACAGGAAAGTAAACAAA
-150 GTCTGCTTCAATGAGATGTTGATCAACAAAAGTTGCAGAAAATATTAGTA
TATTTATCTTTCCATTTCTTGCCAAAACAAATGCCTATAAAAGACCATAC
-50 GCATCCAGACTTTGAAGAATCATCGAATAATCCTCCATTTATCTCCGAAA
1 ATG

P_*NbEAH5*

-350 CTAATCCTTTGTTACTACCAAATTTCTGGCCCCTTAGTTAAGAGTCTTTC
CCGTCCGCAGATTACTAGGAATTTGCCAGAACAGTGATAAAATATTCATA
-250 TTTTGGCTTAAATATTACATCATTATGGTTCTTTTAGTCAGCTGTACTGT
TTTGACCCTTGAGAAAAATTAGTTCCACAGAAAAGTAAACAAAGTCTGCT
-150 TTGATCAGATTTTGAAATATATATATATATATCACTATTGCTTTGTATCT
GCATATTCCATCTCTTGCCAAACCTGCAGCCTATAAAAGAGTATATATAT
-50 GCAACTGAGCTTTGAAGAATACCAACTAACCCTCCCTTTCTCTCCCAATT
1 ATG

P_*NbEAH6*

-350 **TCCCTATTCTCTCTCTATATTGTTCTTATTCTTGCATTATTGTTCTTGTC**
TTTATATTATTTCATAACATGATTAAAATATTAAATAGTTCTCACAAAGT
-250 **TTTGTATTTTTTATAGCCAGCTCAAATATTCCTCTGTCATATTTATTAGTT**
TTGACTAGAAAATTCCCATTTTCTTGAGAAAAATTATTCAATTGTTTAGT
-150 **TTGTTTTCTTCTTTATTAAAATTATGAAAAGCTACAGCTGCCATGGTTGA**
CAGCTGAATAAACAAAGTTGCTGAAAAATGCATCTGTCATTTAAAATGAG
-50 **CATAAATACCCAACAACACTTTGAAGAATCTATCAAACCAAATAACCAAA**
1 **ATG**

SUPPLEMENTARY FIGURE 2 | Nucleotide sequence of promoter region of *NbEAS* and *NbEAH* genes. The 7-bp sequence for the GCC box-like motifs are shown in red letters, 6-bp sequence for the W-box motifs are shown in blue letters, and the start codon of *NbEAS* and *NbEAH* are shown in orange letters. Note that the promoter sequence for *NbEAS10* (-350 to -237) was not available.

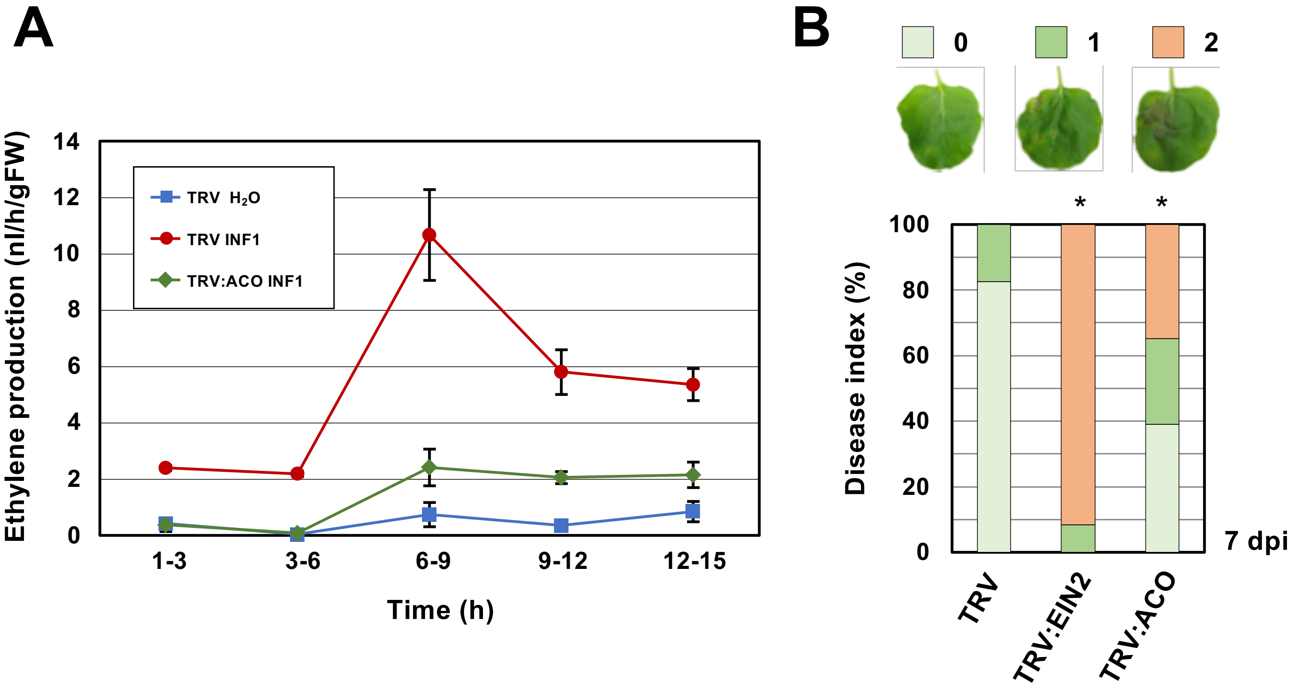

SUPPLEMENTARY FIGURE 3 | (A) Accumulation of ethylene in *N. benthamiana* leaves treated with INF1. Leaves of control (TRV) or *NbACO*-silenced (TRV:ACO) were treated with water (H_2O), or 150 nM INF1 and the amount of ethylene produced was measured by gas chromatography. At least 5 samples from each control and gene-silenced plants were scored for all time points. **(B)** *N. benthamiana* were inoculated with TRV, TRV:EIN2 or TRV:ACO and leaves of control or *NbACO*-silenced plants were inoculated with *P. infestans*. The appearance of disease symptoms was categorized into 3 classes according to the severity of disease symptoms. 0, no visible symptom; 1, small wilted spots in inoculated area; 2, browning >50% of the inoculated side of the leaf. Plot showing percentage of *N. benthamiana* leaves with disease symptom severities represented in the three classes as shown in the upper panels, for leaves of control and gene-silenced plants inoculated with *P. infestans* at 7 days post inoculation (dpi). At least 12 leaves from each control and gene-silenced plants were scored. Data marked with asterisks are significantly different from control as assessed by one-tailed Mann-Whitney U tests: $*P < 0.05$.

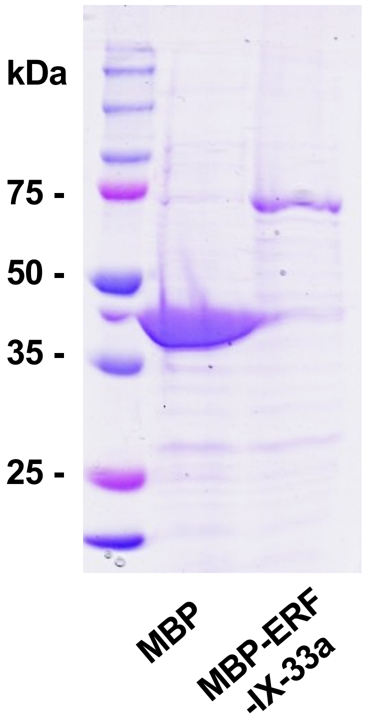

SUPPLEMENTARY FIGURE 4 | Expression and purification of NbERF-IX-33a proteins in *Escherichia coli. E. coli* with pMAL-c5x or pMAL-c5x containing *NbERF-IX-33a* gene were cultured in LB medium with IPTG for the induction of protein expression. Cultured *E. coli* cells were harvested 5 hours after IPTG treatment and MBP (maltose-binding protein) or MBP- NbERF-IX-33a were purified using amylose resins. Eluted fractions were separated by SDS-PAGE and stained with CBB. Protein size markers are shown in kDa. The concentrations of the purified proteins were adjusted and used for the experiments in Figure 8D.

Supplementary Table 1. Plasmids used in this study.

Vector name	Base vector	Restriction site	Insert	Primers used to amplify insert	References	note
**Base vectors**						
pNPP40-GFP	-	-	-	-	Shibata et al. 2016	Base vector for *Agrobacterium* mediated expression of GFP; Kan^R
pBINTRA6	-	-	-	-	Ratcliff et al. 2001	VIGS vector encoding TRV RNA1; Kan^R
pTV00	-	-	-	-	Ratcliff et al. 2001	VIGS vector encoding TRV RNA2 with multi cloning site; Kan^R
pMAL-c5x	-	-	-	-	New England Biolabs	Base vector for the expression of MBP fusion protein in *E. coli* ; Amp^R
pNPP243	pNPP40-GFP	-	-	EGFP-F, pNPP40-R [a]	RIN et al. 2017	Linealized vector for promoter:*GFP* constructs; Kan^R
**Plasmids for promoter analysis**						
pNPP40-INF1	-	-	-	-	Shibata et al. 2016	Vector for *Agrobacterium*- mediated expression of INF1
pNPP40-tGUS	-	-	-	-	RIN et al. 2017	Vector for *Agrobacterium*- mediated expression of truncated *GUS* gene.
pNPP243:P_NbEAS1-400:GFP	pNPP243	Linealized by PCR	P_*NbEAS1-400*	IF_Peas1-400-F, IF_Peas1-R	This study	Expression vector for *GFP* under control of 400-bp *NbEAS1* promoter.
pNPP243:P_NbEAS1-300:GFP	pNPP243	Linealized by PCR	P_*NbEAS1-300*	IF_Peas1-300-F, IF_Peas1-R	This study	Expression vector for *GFP* under control of 300-bp *NbEAS1* promoter.
pNPP243:P_NbEAS1-260:GFP	pNPP243	Linealized by PCR	P_*NbEAS1-260*	IF_Peas1-260-F, IF_Peas1-R	This study	Expression vector for *GFP* under control of 260-bp *NbEAS1* promoter.
pNPP243:P_NbEAS1-230:GFP	pNPP243	Linealized by PCR	P_*NbEAS1-230*	IF_Peas1-230-F, IF_Peas1-R	This study	Expression vector for *GFP* under control of 230-bp *NbEAS1* promoter.
pNPP243:P_NbEAS1-200:GFP	pNPP243	Linealized by PCR	P_*NbEAS1-200*	IF_Peas1-200-F, IF_Peas1-R	This study	Expression vector for *GFP* under control of 200-bp *NbEAS1* promoter.
pNPP243:P_NbEAS4-400:GFP	pNPP243	Linealized by PCR	P_*NbEAS4-400*	IF_Peas4-400-F, IF_Peas4-R	This study	Expression vector for *GFP* under control of 400-bp *NbEAS4* promoter.
pNPP243:P_NbEAS4-300:GFP	pNPP243	Linealized by PCR	P_*NbEAS4-300*	IF_Peas4-300-F, IF_Peas4-R	This study	Expression vector for *GFP* under control of 300-bp *NbEAS4* promoter.
pNPP243:P_NbEAS4-260:GFP	pNPP243	Linealized by PCR	P_*NbEAS4-260*	IF_Peas4-260-F, IF_Peas4-R	This study	Expression vector for *GFP* under control of 260-bp *NbEAS4* promoter.
pNPP243:P_NbEAS4-230:GFP	pNPP243	Linealized by PCR	P_*NbEAS4-230*	IF_Peas4-230-F, IF_Peas4-R	This study	Expression vector for *GFP* under control of 230-bp *NbEAS4* promoter.
pNPP243:P_NbEAS4-200:GFP	pNPP243	Linealized by PCR	P_*NbEAS4-200*	IF_Peas4-200-F, IF_Peas4-R	This study	Expression vector for *GFP* under control of 200-bp *NbEAS4* promoter.
pNPP243:P_NbEAS4-230 (TT):GFP	pNPP243	Linealized by PCR	P_*NbEAS4-230 (TT)*	IF_Peas4-230TT-F, IF_Peas4-R	This study	Expression vector for *GFP* under control of 230-bp *NbEAS4* promoter with mutations in GCC box.
**Plasmids for VIGS**						
pTV00-EIN2	pTV00	-	-	-	Shibata et al. 2010	Silencing vector for *NbEIN2*
pTV00-ACO	pTV00	*Bam* HI/*Sma* I	*NbACO2a* (Partial, 385 bp)	NbACO2-BamHI-F, NbACO2-SmaI-R	This study	Silencing vector for *NbACO* genes
pTV00-NbERF-IX-33	pTV00	*Sma* I	*NbERF-IX-33* (Partial, 300 bp)	IF_pTV00_Nb36116-F, IF_pTV00_Nb36116-R	This study	Silencing vector for *NbERF-IX-33*
pTV00-NbERF-IX-16	pTV00	*Sma* I	*NbERF-IX-16* (Partial, 300 bp)	IF_pTV00_Nb25008-F, IF_pTV00_Nb25008-R	This study	Silencing vector for *NbERF-IX-16*
**Plasmid for protein expression in E. *coli***						
pMAL-NbERF-IX-33a	pMAL-c5x	*Eco* RV	*NbERF-IX-33a*	IF_pMAL-36116-F, IF_pMAL-36116-R	This study	Expression of MBP-NbERF-IX-33a fusion protein in *E. coli*

[a] Primers used to amplify linierlized vector.

Supplementary Table 2. Primers used in this study.

Primer	Sequence	Note
**Primers for construction of expression vectors for promoter analysis**		
EGFP-F	ATGGTGAGCAAGGGCGAGGA	for amplification of pNPP243
pNPP40-R	CTAGATGTAGTTGTAGAATG	for amplification of pNPP243
IF_Peas1-400-F	ACCCTCACTAAAGGGAAATTTCGGCTTCTAATAGT	for construction of pNPP243:P_NbEAS1-400:GFP
IF_Peas1-300-F	ACCCTCACTAAAGGGATAAAGTACATAAACTTTAA	for construction of pNPP243:P_NbEAS1-300:GFP
IF_Peas1-260-F	ACCCTCACTAAAGGGAACAGACCCCAGACGCCAAG	for construction of pNPP243:P_NbEAS1-260:GFP
IF_Peas1-230-F	ACCCTCACTAAAGGGTAGGCTGCTGGTTGGCTGAC	for construction of pNPP243:P_NbEAS1-230:GFP
IF_Peas1-200-F	ACCCTCACTAAAGGGGTGTAAAGTCAAGTAAGGCA	for construction of pNPP243:P_NbEAS1-200:GFP
IF_Peas1-R	GCCCTTGCTCACCATTATATTGCTAATAAATTAAA	for construction of pNPP243:P_NbEAS1:GFP
IF_Peas4-400-F	ACCCTCACTAAAGGGGAAAGTTTTCAAATATCACT	for construction of pNPP243:P_NbEAS4-400:GFP
IF_Peas4-300-F	ACCCTCACTAAAGGGATTTTAGAGACGTAGGATGT	for construction of pNPP243:P_NbEAS4-300:GFP
IF_Peas4-260-F	ACCCTCACTAAAGGGATGTAAGAAATACTGCTCCT	for construction of pNPP243:P_NbEAS4-260:GFP
IF_Peas4-230-F	ACCCTCACTAAAGGGATCAATTGAGACGCCAAGAA	for construction of pNPP243:P_NbEAS4-230:GFP
IF_Peas4-200-F	ACCCTCACTAAAGGGAGACGCCAAGGAAGAAATAT	for construction of pNPP243:P_NbEAS4-200:GFP
IF_Peas4-230TT-F	ACCCTCACTAAAGGGATCAATTGATACTCCAAGAA	for construction of pNPP243:P_NbEAS4-230TT:GFP
IF_Peas4-R	GCCCTTGCTCACCATTGCTATAGAATTTATACTCT	for construction of pNPP243:P_NbEAS4:GFP
**Primers for construction of VIGS vectors**		
NbACO2-BamHI-F	TACATAGGATCCTGGAGAGTTTCCCAGTGG	for construction of pTV00-ACO
NbACO2-SmaI-R	CAGAGCCCCGGGAAAAGTTGCTCTGCTAAT	for construction of pTV00-ACO
IF_pTV00_Nb36116-F	ACTAGTGGATCCCCCGATGAAGAAGCTGCAATT	for construction of pTV00-NbERF-IX-33
IF_pTV00_Nb36116-R	GAATTCCTGCAGCCCTTAACTGACTATTAATTG	for construction of pTV00-NbERF-IX-33
IF_pTV00_Nb25008-F	ACTAGTGGATCCCCCCAGGTCATGGGAAAGAAG	for construction of pTV00-NbERF-IX-16
IF_pTV00_Nb25008-R	GAATTCCTGCAGCCCCTAGGTCCTGTCATAACC	for construction of pTV00-NbERF-IX-16
**Primers for qRT-PCR**		
RT-GUS-F	TCAAAAAACTCGACGGCCTG	for qRT-PCR of tGUS
RT-GUS-R	TTCGGTATAAAGACTTCGCG	for qRT-PCR of tGUS
RT-GFP-F	CAACTACAACAGCCACAACG	for qRT-PCR of GFP
RT-GFP-R	TCTTTGCTCAGGGCGGACTG	for qRT-PCR of GFP
**Primers for construction of protein expression vector**		
IF_pMAL-36116-F	ATGGGCGGCCGCGATATGAATCATTCTATTTATAC	for construction of pMAL-NbERF-IX-33a
IF_pMAL-36116-R	GGATCCGTCGACGATTTAACTGACTATTAATTGAT	for construction of pMAL-NbERF-IX-33a

Extension sequences for In-fusion reaction are in red letters.

Mismatches to introduce mutations are highlighted in blue letters.

Restriction sites used for the construction of vectors are underlined.

Supplementary Table 3. *Nicotiana benthamiana* genes categorized in cluster 2.

Gene ID*	Expression (FPKM value)									Annotation (BlastX)
	0 h (Control)	H_2O				150 nM INF1				
		3 h	6 h	12 h	24 h	3 h	6 h	12 h	24 h	
Niben101Scf01084g05013	89.89	186.30	368.21	600.92	472.93	247.88	508.01	1076.55	1194.83	Glycine-rich protein 3-like [*N. tabacum*]
Niben101Ctg13380g00001	385.51	364.03	550.20	828.68	578.02	521.03	865.15	1149.75	1027.51	Peptidyl-prolyl cis-trans isomerase [*N. sylvestris*]
Niben101Scf00428g14006	474.80	275.27	518.21	548.99	594.95	152.92	330.73	314.27	885.58	Lysine-rich arabinogalactan protein 19-like [*N. sylvestris*]
Niben101Scf01084g03004	103.60	123.50	202.01	202.13	176.49	189.34	477.48	786.50	513.54	Glycine-rich protein-like [*N. sylvestris*]
Niben101Scf00897g03002	87.50	56.10	142.58	136.14	117.91	360.45	591.60	416.94	511.91	Glutathione S-transferase [*N. sylvestris*]
Niben101Scf25768g00021	55.89	53.63	105.20	87.05	73.63	131.45	244.79	340.24	401.41	Protein RALF-like 27 [*N. tabacum*]
Niben101Scf00646g07010	48.09	142.16	249.09	289.19	104.00	484.94	741.33	461.93	389.30	Uncharacterized protein LOC104227479 [*N. sylvestris*]
Niben101Scf02771g01007	99.13	133.84	156.75	122.69	119.63	228.76	295.33	232.14	377.09	Heat shock cognate 70 kDa protein 2-like [*N. attenuata*]
Niben101Scf06590g00003	96.07	104.47	170.95	177.97	188.18	131.28	214.77	201.22	362.39	L-ascorbate peroxidase 2, cytosolic [*N. tabacum*]
Niben101Scf10505g00007	69.73	44.34	79.57	106.93	54.91	93.57	271.58	225.90	342.59	Protein disulfide-isomerase-like [*N. tomentosiformis*]
Niben101Scf13188g00012	46.14	49.60	118.23	204.73	165.49	75.52	135.37	267.48	305.54	Abscisic stress-ripening protein 2 [*N. sylvestris*]
Niben101Scf01048g00008	188.71	83.96	128.06	288.16	247.34	83.87	129.00	421.04	292.60	Glycine-rich domain-containing protein 1-like [*N. tabacum*]
Niben101Scf02886g01003	85.05	58.15	115.12	139.94	110.30	93.31	229.52	335.41	286.02	Metallothionein-like protein type 2 [*N. attenuata*] *
Niben101Scf04364g01014	72.58	96.56	108.35	88.80	89.59	155.92	208.27	157.23	279.79	Heat shock cognate 70 kDa protein 2-like [*N. attenuata*]
Niben101Scf03052g00011	107.10	82.41	101.34	196.07	165.30	85.40	160.23	319.47	278.98	Glycine-rich protein-like [*N. sylvestris*]
Niben101Scf00447g02008	61.17	72.22	98.31	84.84	75.39	129.86	148.76	106.96	260.77	Eukaryotic translation initiation factor 1A-like [*N. tomentosiformis*]
Niben101Scf02230g01003	59.86	55.93	74.09	76.65	64.64	80.33	105.32	142.47	258.76	Leucine-rich repeat protein 1-like [*N. tomentosiformis*]
Niben101Scf01445g00016	81.09	35.38	89.26	168.54	45.05	78.21	178.18	307.02	255.86	Cysteine proteinase 3 precursor [*N. tabacum*]
Niben101Scf04209g01002	162.33	124.74	258.54	233.77	75.35	247.61	662.72	811.22	248.52	Uncharacterized protein LOC109213470 [*N. attenuata*]
Niben101Scf07459g00017	93.61	83.09	96.49	74.88	82.39	108.76	225.24	136.32	239.18	Phosphomannomutase [*N. tabacum*]
Niben101Scf06195g00002	71.82	71.44	101.03	110.29	100.23	97.39	144.20	156.80	214.46	L-ascorbate peroxidase 2, cytosolic [*N. tabacum*]
Niben101Scf05152g01001	46.47	23.08	65.72	80.39	35.08	21.61	83.74	120.30	209.42	Cytochrome b561 and DOMON domain-containing protein [*N. sylvestris*]
Niben101Scf01365g05003	124.04	85.94	146.82	173.34	127.48	159.98	400.55	383.10	205.56	Sucrose transporter 1-1 [*N. tabacum*]
Niben101Scf06726g00034	132.48	88.13	163.55	284.80	170.43	78.64	263.36	464.14	204.99	BURP domain protein USPL1-like [*N. tomentosiformis*]
Niben101Scf00578g05007	20.47	22.40	33.65	28.81	28.82	35.65	182.73	113.98	203.12	Calcium-binding protein PBP1-like [*N. sylvestris*]
Niben101Scf03823g01005	99.70	49.02	115.17	168.62	96.83	64.64	181.33	173.01	203.12	Pectinesterase/pectinesterase inhibitor U1 [*N. attenuata*]
Niben101Scf05340g00009	65.13	66.31	87.84	104.41	70.41	82.35	118.49	142.08	201.43	Uncharacterized protein LOC109212171 [*N. attenuata*]
Niben101Scf00983g02001	104.98	93.37	129.98	196.45	99.13	196.76	316.33	383.35	196.22	Isocitrate dehydrogenase [NADP] [*N. tomentosiformis*]
Niben101Scf16258g02004	17.98	49.15	98.45	183.09	166.74	58.12	146.27	190.12	190.14	Defensin J1-2 [*N. attenuata*]
Niben101Scf01032g00006	155.41	51.44	75.47	141.24	115.99	65.34	128.11	254.36	184.82	Dihydroflavonol-4-reductase-like [*Capsicum annuum*]
Niben101Scf02319g12008	44.63	62.15	142.00	167.34	58.37	165.06	326.62	289.52	176.93	BURP domain protein RD22-like [*N. tomentosiformis*]
Niben101Scf01008g03019	126.96	43.86	114.68	128.05	115.01	33.33	103.93	122.64	173.20	Homeobox protein 2-like [*N. attenuata*]
Niben101Scf37733g00002	37.21	40.31	24.25	77.09	108.03	29.12	45.62	82.44	169.55	5'-adenylylsulfate reductase 1, chloroplastic-like [*N. tabacum*]
Niben101Scf04174g01004	42.69	51.49	81.87	63.01	35.57	156.94	201.60	176.25	160.52	Glutathione S-transferase [*N. attenuata*]
Niben101Scf04140g00003	66.27	42.60	71.56	88.99	86.85	130.40	161.65	289.30	158.74	Uncharacterized protein LOC104227479 [*N. sylvestris*]
Niben101Scf00345g01021	73.47	25.51	61.11	156.47	108.70	29.74	69.97	153.50	151.19	Glutamine synthetase isoform X1 [N. sylvestris]
Niben101Scf00215g02009	58.49	44.93	64.45	79.24	52.86	74.39	95.53	125.93	143.89	Leucine-rich repeat protein 1-like [*N. tomentosiformis*]
Niben101Scf02508g01012	33.94	44.83	63.71	55.96	47.11	74.77	95.84	82.31	130.22	Small acidic protein 1 [*Solanum tuberosum*]
Niben101Scf05442g03015	48.76	32.51	42.00	50.91	54.13	31.66	69.33	137.25	127.96	Phenylalanine ammonia-lyase [*N. tomentosiformis*]
Niben101Scf06890g01022	12.39	22.97	40.71	17.77	16.09	64.87	100.34	75.55	126.57	Endoplasmin homolog [*N. attenuata*]
Niben101Scf18771g00012	23.30	39.70	48.06	31.87	54.34	44.17	132.75	59.77	124.88	Heme-binding-like protein, chloroplastic [*N. sylvestris*]
Niben101Scf03460g04004	10.17	11.28	41.10	59.91	46.00	13.47	72.99	94.26	124.70	Lignin-forming anionic peroxidase [*N. tomentosiformis*]
Niben101Scf02222g01001	28.17	30.90	50.12	47.74	32.53	47.75	75.04	76.39	118.67	Uncharacterized protein LOC109212171 [*N. attenuata*]
Niben101Scf01025g02004	63.19	42.64	52.19	80.93	83.61	71.18	178.45	184.97	116.54	Arginine decarboxylase-like [*N. attenuata*]
Niben101Scf01520g04005	121.86	41.25	68.33	121.99	84.75	47.41	97.89	183.82	116.02	Stem-specific protein TSJT1 [*N. sylvestris*]
Niben101Scf03425g00005	67.70	35.73	46.07	73.70	55.07	45.82	92.22	172.05	115.06	Sucrose transporter 1-1 [*N. tabacum*]
Niben101Scf08341g01001	48.05	37.80	52.26	76.23	68.23	48.29	77.60	74.02	113.28	GTP-binding nuclear protein Ran-B1 [*N. tabacum*]
Niben101Scf07253g02012	26.46	37.12	58.03	55.75	33.51	56.42	94.60	72.70	110.58	Microsomal glutathione S-transferase 3 [*N. tomentosiformis*]
Niben101Scf00837g11004	14.74	23.75	47.37	22.54	17.49	58.95	112.53	59.95	109.15	Endoplasmin homolog [*N. attenuata*]
Niben101Scf00107g03016	9.55	17.72	33.46	21.41	9.64	44.75	73.71	58.79	106.56	Kynurenine formamidase-like [*N. tabacum*]

*Top 50 genes highly expressed in INF1-treated leaves 24 after the treatment.

Supplementary Table 4. *Nicotiana benthamiana* genes categorized in cluster 4.

Gene ID*	Expression (FPKM value)									Annotation (BlastX)
	0 h	H_2O				150 nM INF1				
	(Control)	3 h	6 h	12 h	24 h	3 h	6 h	12 h	24 h	
Niben101Scf06525g03013	22.02	46.50	112.94	125.94	87.30	231.76	386.77	728.54	1805.14	NbSAR8.2m gene product [*N. benthamiana*]
Niben101Scf02041g00002	4.05	35.41	33.97	29.04	16.17	65.65	68.15	173.15	1291.63	Acidic chitinase PR-Q [*N. tabacum*]
Niben101Scf12789g00006	0.94	5.04	22.55	30.87	28.06	95.08	239.38	616.75	760.73	Cysteine-rich repeat secretory protein 55-like [*N. tabacum*]
Niben101Scf10834g03005	23.16	32.45	61.18	40.45	25.38	89.88	179.66	210.68	409.89	Calreticulin-3 NbCRT3a2 [*N. benthamiana*]
Niben101Scf01400g00014	3.65	3.18	2.65	2.29	3.61	2.98	2.00	6.50	399.79	Pathogenesis-related protein R minor form [*N. sylvestris*]
Niben101Scf01719g08010	6.20	11.49	5.22	7.81	5.29	28.79	44.42	171.94	215.06	PDR-type ACB transporter NbPDR2a [*N. benthamiana*]
Niben101Scf00414g07005	16.81	22.84	24.80	23.02	21.83	127.01	150.73	235.03	210.88	Farnesyl pyrophosphate synthase NbFPPS1b [*N. benthamiana*]
Niben101Scf10055g07005	12.17	27.62	24.07	14.25	13.98	81.54	89.96	182.21	207.87	NADP-dependent malic enzyme [*N. sylvestris*]
Niben101Scf06256g01001	18.72	18.53	33.23	22.98	17.01	48.16	73.74	123.27	195.57	Calreticulin-3 NbCRT3a1 [*N. benthamiana*]
Niben101Scf01051g09003	9.76	7.27	7.28	12.04	12.31	8.28	10.37	49.70	180.33	FAM10 family protein [*N. attenuata*]
Niben101Scf00213g01002	3.05	4.67	12.01	8.80	5.68	24.84	60.17	116.48	163.47	G-type lectin S-receptor-like serine/threonine-protein kinase RLK1 [*N. sylvestris*]
Niben101Scf02636g05003	1.18	2.44	1.97	1.19	1.91	4.30	5.00	59.29	158.80	Uncharacterized protein LOC107818746 [*N. tabacum*]
Niben101Scf03990g00010	4.50	3.89	9.46	16.69	5.66	13.11	67.90	114.79	154.67	Peroxidase 21 [*N. sylvestris*]
Niben101Scf14996g00009	4.08	3.71	1.56	2.06	2.42	3.11	2.62	20.86	149.69	Catalase isozyme 1 [*N. sylvestris*]
Niben101Scf08130g00014	1.06	2.46	2.37	1.17	1.05	6.13	10.12	26.12	141.70	Glutathione S-transferase [*N. attenuata*]
Niben101Scf05283g00016	7.62	14.52	21.35	15.58	11.48	29.88	68.00	88.02	129.93	Carboxylesterase 17 [*N. attenuata*]
Niben101Scf04941g01011	3.28	2.92	5.41	4.39	4.30	14.29	13.67	39.57	127.61	LysM domain receptor-like kinase 4 [*N. sylvestris*]
Niben101Scf02111g16018	12.24	13.05	14.87	16.25	14.79	25.36	46.74	129.36	127.43	ATP-citrate synthase beta chain protein 2 [*N. sylvestris*]
Niben101Scf08510g01006	1.46	7.71	6.36	5.90	4.40	48.53	78.68	201.24	120.12	Heavy metal-associated isoprenylated plant protein 39-like [*N. attenuata*]
Niben101Scf02786g03004	0.77	8.29	10.83	3.79	1.24	48.18	46.50	64.95	117.52	Uncharacterized protein LOC104218427 [*N. sylvestris*]
Niben101Scf03147g10015	8.85	3.73	8.71	2.24	3.77	4.36	13.27	33.80	105.04	Glutathione S-transferase [*N. attenuata*]
Niben101Scf02195g01001	4.10	4.37	5.56	24.94	10.59	7.60	39.67	92.75	102.70	Calcium-binding protein CML41 [*N. sylvestris*]
Niben101Scf03816g01001	4.48	5.00	7.21	7.66	5.62	26.67	38.31	76.94	101.82	LRR receptor-like serine/threonine/tyrosine-protein kinase SOBIR1 [*N. tabacum*]
Niben101Scf02658g01008	1.66	11.01	13.00	5.17	3.63	37.10	60.20	48.98	97.14	Dirigent protein 22-like [*N. tabacum*]
Niben101Scf08939g02004	0.74	1.50	0.94	1.30	1.43	1.55	1.56	18.77	96.15	Hypothetical protein [*N. attenuata*]
Niben101Scf03015g03010	7.54	15.24	14.06	11.68	9.16	25.39	21.97	69.36	94.56	14-3-3-like protein E [*N. tabacum*]
Niben101Scf01111g01003	0.96	4.50	2.85	2.22	1.45	71.49	45.95	124.19	93.74	3-hydroxy-3-methylglutaryl-CoA synthase NbHMGR1a [*N. benthamiana*]
Niben101Ctg16217g00002	1.80	2.85	2.55	2.33	2.10	20.76	20.89	30.95	92.37	Uncharacterized protein LOC109221780 [*N. attenuata*]
Niben101Scf05601g00010	1.08	3.82	6.41	6.89	1.96	31.14	47.02	72.24	91.65	Sugar transport protein 13 [*N. attenuata*]
Niben101Scf09846g01010	2.57	3.76	8.89	4.40	9.05	34.70	54.04	48.75	87.76	Quinone-oxidoreductase homolog, chloroplastic [*N. sylvestris*]
Niben101Scf01742g05006	4.10	3.94	4.22	3.78	4.54	14.83	22.37	67.11	81.92	ATP-citrate synthase alpha chain protein 2 [*N. sylvestris*]
Niben101Scf05872g02002	6.41	5.26	11.69	13.60	20.42	4.51	14.18	14.84	80.91	Dirigent protein 22-like [*N. attenuata*]
Niben101Scf08940g01001	0.71	8.52	6.24	1.79	0.87	36.15	37.28	68.50	74.60	Lysine histidine transporter 1-like [*N. sylvestris*]
Niben101Scf10735g00016	7.96	5.89	8.69	22.11	18.46	7.16	17.58	54.43	72.74	Pathogenesis-related protein STH-2-like [*N. attenuata*]
Niben101Scf10336g01001	1.76	1.44	2.60	3.38	3.48	5.63	63.99	54.61	72.12	Metalloendoproteinase 1-like [*N. sylvestris*]
Niben101Scf05060g07008	0.06	3.24	2.89	1.86	0.67	6.92	108.07	31.76	68.41	Calcium-binding protein CML46 [*N. tomentosiformis*]
Niben101Scf02171g00008	5.65	5.57	3.34	4.61	5.75	9.60	41.70	84.12	66.46	Wound-inuduced protein kinase [*N. benthamiana*]
Niben101Scf02349g03002	2.20	3.18	3.12	2.72	1.56	4.71	6.87	16.06	66.32	Suberization-associated anionic peroxidase-like [*N. tabacum*]
Niben101Scf02063g05001	1.54	1.53	0.75	2.79	1.77	1.55	6.87	27.40	63.16	Ethylene-responsive transcription factor 1B-like [*N. attenuata*]
Niben101Scf01015g01002	0.35	0.40	0.67	0.50	0.78	0.50	1.01	9.87	62.80	Wound-induced protein WIN1-like [*N. sylvestris*]
Niben101Scf02622g07001	3.08	2.07	1.90	2.08	3.09	6.29	15.38	40.40	61.44	Glycosyltransferase family protein 64 protein C5-like [*N. attenuata*]
Niben101Scf08020g06001	0.81	5.66	4.06	2.04	1.41	14.14	58.08	32.91	59.25	Harpin inducing protein [*N. tabacum*]
Niben101Scf04015g01004	0.28	0.73	0.39	1.19	1.71	2.86	3.34	24.19	59.10	Phosphoglycerate mutase-like protein AT74H [*N. tomentosiformis*]
Niben101Scf02639g03015	3.70	7.54	4.41	5.55	5.38	13.45	44.18	42.02	58.78	ALA-interacting subunit 1-like [*N. attenuata*]
Niben101Scf03114g02002	2.62	4.64	4.37	5.50	8.01	13.91	38.47	55.30	58.48	Heavy metal-associated isoprenylated plant protein 39-like [*N. attenuata*]
Niben101Scf01574g09001	1.39	3.74	3.68	3.27	1.76	11.21	55.25	50.41	58.32	Uncharacterized protein LOC104235457 [*N. sylvestris*]
Niben101Scf02072g02017	9.11	4.71	7.86	13.01	9.46	6.11	10.56	38.39	58.01	Spermidine coumaroyl-CoA acyltransferase [*N. tomentosiformis*]
Niben101Scf05378g01017	6.52	2.59	10.84	9.79	4.88	17.18	21.20	35.65	57.95	Uncharacterized protein LOC104240896 [*N. sylvestris*]
Niben101Scf00225g00003	2.09	8.32	3.16	3.27	4.82	12.81	5.29	32.70	57.38	Flavanone 3-dioxygenase [*N. sylvestris*]
Niben101Scf00700g09001	3.44	13.07	12.96	7.05	4.15	23.71	28.36	27.08	56.92	Caffeic acid 3-O-methyltransferase-like [*N. attenuata*]

*Top 50 genes highly expressed in INF1-treated leaves 24 after the treatment.

Supplementary Table 5. *Nicotiana benthamiana* genes categorized in cluster 10.

Gene ID*	Expression (FPKM value)									Annotation (BlastX)
	0 h (Control)	H_2O				150 nM INF1				
		3 h	6 h	12 h	24 h	3 h	6 h	12 h	24 h	
Niben101Scf02543g01008	12.44	153.43	100.46	13.32	6.30	448.61	268.75	195.65	546.31	1-aminocyclopropane-1-carboxylate oxidase NbACO1a [*N. benthamiana*]
Niben101Scf13429g03011	0.19	42.41	128.05	7.14	1.40	412.72	625.15	232.46	110.86	14 kDa proline-rich protein DC2.15-like [*N. attenuata*]
Niben101Scf08039g01005	0.46	28.97	13.08	1.91	1.04	163.21	74.73	51.93	103.35	1-aminocyclopropane-1-carboxylate oxidase NbACO1b [*N. benthamiana*]
Niben101Scf07103g01015	30.63	23.82	177.94	33.97	0.00	23.38	80.96	22.93	87.86	ATP synthase CF1 epsilon subunit [*N. tabacum*]
Niben101Scf00148g00004	0.94	21.83	14.87	1.00	1.10	41.15	82.72	62.45	75.85	21 kDa protein-like [*N. tabacum*]
Niben101Scf05279g05006	0.34	20.97	7.76	1.01	0.41	39.81	41.60	43.19	37.01	Uncharacterized protein LOC104231083 [*N. sylvestris*]
Niben101Scf01236g05023	15.21	23.39	22.59	20.52	21.26	24.72	0.00	30.89	31.88	Hypothetical protein Lal_00000388 [*Lupinus albus*]
Niben101Scf01640g03006	0.23	58.93	7.90	0.72	0.48	79.67	9.54	10.33	28.87	hypothetical protein A4A49_07063 [*N. attenuata*]
Niben101Scf07829g01008	0.51	20.59	2.44	0.57	0.17	51.84	48.36	29.95	27.43	uncharacterized protein LOC104247702 [*N. sylvestris*]
Niben101Ctg15035g00004	0.04	4.70	4.56	0.41	0.28	53.87	26.39	36.10	25.73	CASP-like protein PIMP1 [*N. attenuata*]
Niben101Scf03861g00008	0.05	1.92	0.20	0.21	0.11	6.95	12.04	10.89	16.82	Peroxidase 4 [*Capsicum chinense*]
Niben101Scf05326g00010	0.19	3.51	3.03	0.48	0.35	8.02	9.15	21.07	14.71	Lipoxygenase homology domain-containing protein 1-like [*N. tabacum*]
Niben101Scf00109g07005	0.00	1.36	0.39	0.03	0.04	1.06	4.46	9.35	12.93	Calcium-binding protein CML19 [*N. attenuata*]
Niben101Scf00755g00004	8.66	6.30	9.69	4.36	0.00	5.35	12.22	4.48	12.65	Unknown protein
Niben101Scf03647g03023	0.21	0.22	0.00	0.10	0.13	0.12	0.07	0.40	11.33	Uncharacterized protein LOC104118395 [*N. tomentosiformis*]
Niben101Scf00390g03001	0.13	2.72	0.57	0.23	0.07	3.06	0.70	3.49	9.79	Proline dehydrogenase 2, mitochondrial-like [*N. tabacum*]
Niben101Scf21484g01002	4.54	1.21	4.23	6.74	3.96	0.00	6.62	1.83	9.15	Uncharacterized protein LOC104099480 [*N. tomentosiformis*]
Niben101Scf00050g01002	1.76	5.55	1.39	0.38	2.07	2.98	2.33	2.34	7.85	Phylloplanin-like [*N. tabacum*]
Niben101Scf05118g11009	0.00	0.94	0.56	0.01	0.03	3.21	1.29	2.51	6.08	E3 ubiquitin-protein ligase Hakai-like [*N. attenuata*]
Niben101Scf02613g05004	0.06	1.32	0.29	0.00	0.00	2.38	0.97	0.87	6.08	EG45-like domain containing protein [*N. tabacum*]
Niben101Scf06578g00021	4.86	1.54	2.51	2.43	2.81	0.22	2.03	2.90	5.84	Calcium-binding protein PBP1-like [*N. sylvestris*]
Niben101Scf07751g00003	0.19	2.65	0.84	0.28	0.03	7.94	6.27	6.11	5.73	AT-hook motif nuclear-localized protein 17-like [*N. attenuata*]
Niben101Scf09078g00001	1.65	0.81	0.60	0.50	0.71	0.03	0.48	1.15	5.07	Uncharacterized protein LOC108943634 [*N. tomentosiformis*]
Niben101Scf01376g04034	0.09	6.37	1.48	0.11	0.02	9.41	4.70	3.45	5.02	Late blight resistance protein homolog R1B-16 isoform X2 [*N. tabacum*]
Niben101Scf02085g12016	0.01	2.20	0.25	0.11	0.01	13.05	2.98	3.36	4.48	L-type lectin-domain containing receptor kinase S.4-like [*N.* attenuata]
Niben101Scf01196g00008	1.67	1.58	0.00	3.28	2.74	0.31	0.74	0.71	4.22	Unknown protein
Niben101Scf00372g02011	0.01	1.36	0.07	0.09	0.03	7.28	7.62	5.52	4.11	uncharacterized protein DDB_G0271670-like [*N. attenuata*]
Niben101Ctg10923g00001	0.24	0.31	0.21	0.29	0.24	0.19	0.02	0.81	3.87	vacuolar-sorting receptor 6-like [*N. tomentosiformis*]
Niben101Scf05434g00011	3.25	3.59	1.06	1.07	0.00	14.44	4.78	4.88	3.79	Sulfate transporter 3.3 [*N. attenuata*]
Niben101Scf01451g05015	2.19	1.05	0.00	2.29	1.35	0.52	1.35	2.31	3.74	oligoribonuclease-like [*N. attenuata*]
Niben101Scf02191g03003	0.15	0.33	0.00	0.32	2.33	0.13	0.12	0.24	3.74	hypothetical protein H5410_023541 [*Solanum commersonii*]
Niben101Scf08718g00004	6.76	0.35	1.23	0.93	0.08	1.51	2.96	3.62	2.80	Unknown protein
Niben101Scf01212g01017	0.75	1.81	2.71	1.34	1.17	0.17	1.56	1.84	2.76	photosystem II protein D1/D2 superfamily [*Helianthus annuus*]
Niben101Scf00484g03003	1.22	0.76	1.21	1.43	1.33	1.46	0.00	1.76	2.70	serine/arginine-rich SC35-like splicing factor SCL33 isoform X2 [*N. attenuata*]
Niben101Scf02907g06004	0.02	1.99	0.80	0.00	0.95	3.76	2.31	0.47	2.43	8-hydroxygeraniol dehydrogenase-like [*N. tomentosiformis*]
Niben101Scf03213g01009	1.09	0.52	0.05	0.58	0.60	0.36	0.39	0.97	1.87	hypothetical protein EJD97_005407 [*S. chilense*]
Niben101Scf02060g00010	0.15	0.13	0.24	0.21	0.00	0.30	0.23	0.70	1.78	MADS-box transcription factor 23-like [*N. attenuata*]
Niben101Scf08465g03014	0.34	0.44	0.00	0.17	0.30	0.28	0.08	0.08	1.72	GDSL esterase/lipase At5g37690 [*N. sylvestris*]
Niben101Scf10492g00004	0.08	0.08	0.06	0.10	0.13	0.00	0.08	0.46	1.69	Glycosyltransferase At5g25310 [*N. attenuata*]
Niben101Scf09710g00026	0.65	0.60	1.31	2.00	0.78	0.58	0.11	1.01	1.57	Hypothetical protein NitaMp145 [*N. tabacum*]
Niben101Scf02869g18005	0.12	0.24	0.19	0.00	0.00	0.27	0.08	0.37	1.54	Elicitor-responsive protein 1-like [*N. sylvestris*]
Niben101Scf00428g16011	0.47	0.42	0.41	0.16	0.63	1.47	1.08	0.00	1.51	V-type proton ATPase subunit d2 [*N. sylvestris*]
Niben101Scf01517g08025	0.40	0.83	0.70	1.82	0.72	0.00	1.72	1.38	1.44	Uncharacterized protein LOC104232423 [*N. sylvestris*]
Niben101Scf07792g00002	0.28	0.49	1.37	0.42	0.52	0.00	1.72	0.33	1.41	Vicilin-like seed storage protein [*N. attenuata*]
Niben101Scf04731g11004	0.02	0.25	0.02	0.00	0.02	0.06	0.03	0.06	1.26	Citrate-binding protein-like [*N. tabacum*]
Niben101Scf03886g04003	0.92	0.48	0.45	1.18	0.54	0.04	0.56	1.03	1.15	HVA22-like protein e [*N. tabacum*]
Niben101Scf02085g12014	0.06	13.99	0.77	0.05	0.01	29.16	1.91	0.96	1.07	E3 ubiquitin-protein ligase Praja-2-like [*N. sylvestris*]
Niben101Scf06517g00020	0.30	0.25	0.00	0.15	0.13	0.25	0.11	0.37	1.00	MADS-box transcription factor 23-like isoform X1 [*N. attenuata*]
Niben101Scf01111g03001	0.03	0.03	0.05	0.00	0.00	0.03	0.04	0.15	0.99	MATE efflux family protein 5-like [*N. sylvestris*]
Niben101Scf04981g00001	0.02	0.22	0.08	0.00	0.03	0.23	0.11	1.20	0.96	Glutathione transferase GST 23-like [*N. sylvestris*]
Niben101Scf03422g04036	0.02	1.05	0.07	0.00	0.09	5.04	0.98	2.11	0.93	Plant cadmium resistance 8-like [*N. tomentosiformis*]

*Top 50 genes highly expressed in INF1-treated leaves 24 after the treatment.

Supplementary Table 6. *Nicotiana benthamiana* genes categorized in cluster 14.

Gene ID*	Expression (FPKM value)									Annotation (BlastX)
	0 h (Control)	H_2O				150 nM INF1				
		3 h	6 h	12 h	24 h	3 h	6 h	12 h	24 h	
Niben101Scf08921g02024	0.47	0.45	2.92	3.21	1.98	3.83	11.26	181.07	963.96	Zingipain-2-like [*N. attenuata*]
Niben101Scf03385g02011	1.98	4.01	4.21	2.75	2.62	39.14	52.84	211.72	690.42	Uncharacterized protein LOC104232799 [*N. sylvestris*]
Niben101Ctg13736g00004	0.38	0.22	0.40	0.70	5.90	2.86	3.69	86.46	672.36	Glucan endo-1,3-beta-glucosidase, acidic isoform GI9-like [*N. sylvestris*]
Niben101Scf01001g00003	0.27	0.16	0.27	0.71	5.81	2.74	3.55	76.80	626.78	Glucan endo-1,3-beta-glucosidase, acidic isoform GI9-like [*N. sylvestris*]
Niben101Scf09740g00003	0.18	0.06	0.27	2.17	5.74	0.13	0.43	19.50	600.88	Uncharacterized protein LOC107783307 [*N. tabacum*]
Niben101Scf01001g00005	0.26	0.12	0.25	0.74	5.36	2.67	3.21	73.94	570.31	Glucan endo-1,3-beta-glucosidase, acidic isoform gi9 [*N. attenuata*]
Niben101Scf00107g03008	0.25	0.03	0.14	0.50	5.27	0.16	0.55	28.48	569.39	Pathogenesis-related protein 1B [*N. tabacum*]
Niben101Scf03930g00018	0.97	26.93	14.27	3.22	1.74	214.84	83.54	192.51	566.23	Cucumber peeling cupredoxin-like [*N. sylvestris*]
Niben101Scf02407g03010	1.56	5.27	5.21	4.44	3.72	34.22	48.64	201.52	560.15	Uncharacterized protein LOC109240683 [*N. attenuata*]
Niben101Scf01001g00004	0.19	0.14	0.24	0.70	4.98	2.44	3.02	68.19	530.15	Glucan endo-1,3-beta-glucosidase, acidic isoform GI9-like [*N. sylvestris*]
Niben101Scf00640g04023	0.00	0.73	0.73	0.79	0.35	9.14	17.16	86.86	434.56	Bifunctional epoxide hydrolase 2-like [*N. tomentosiformis*]
Niben101Scf01084g03003	0.08	0.71	1.94	0.22	0.12	13.73	31.94	96.53	374.31	NbSAR8.2d gene product [*N. benthamiana*]
Niben101Scf01934g02004	0.04	0.09	0.05	0.09	0.14	0.60	4.93	131.70	334.61	Glucan endo-1,3-beta-glucosidase, basic vacuolar isoform GGIB50 [*N. sylvestris*]
Niben101Scf02877g02005	0.12	0.06	0.28	0.58	1.03	2.96	26.29	124.35	324.87	Strictosidine synthase 1-like [*N. sylvestris*]
Niben101Scf02203g05002	1.33	6.71	4.77	2.78	1.69	82.45	94.85	222.74	295.51	3-hydroxy-3-methylglutaryl-coenzyme A reductase NbHMGR2 [*N. benthamiana*]
Niben101Scf02819g00005	0.70	4.95	7.08	2.14	1.49	58.05	53.59	109.74	280.57	Cupredoxin-like [*N. tomentosiformis*]
Niben101Scf04869g03002	0.34	0.21	0.51	0.68	2.07	3.66	6.26	56.88	276.55	Glucan endo-1,3-beta-glucosidase, acidic isoform GL161 [*N. sylvestris*]
Niben101Scf02918g00003	0.31	0.53	0.97	0.74	0.55	11.06	22.61	202.69	275.20	1-aminocyclopropane-1-carboxylate oxidase NbACO4 [*N. benthamiana*]
Niben101Scf35444g00004	0.56	5.66	5.06	3.01	2.47	19.32	27.38	81.16	263.68	Glutathione S-transferase [*N. sylvestris*]
Niben101Scf10986g00001	0.73	1.38	2.20	3.17	4.16	11.74	21.50	142.36	219.62	Cysteine-rich repeat secretory protein 55-like [*N. tabacum*]
Niben101Scf03993g05005	0.09	6.12	4.35	0.57	0.25	305.27	189.22	239.25	211.45	5-*epi*-aristolochene synthase NbEAS4 [*N. benthamiana*]
Niben101Scf04053g02006	0.03	0.00	0.00	0.04	0.08	0.01	0.24	21.12	206.72	Basic form of pathogenesis-related protein 1-like [*N. sylvestris*]
Niben101Scf10488g01001	0.97	2.24	1.70	0.69	0.91	6.16	8.04	52.83	204.46	Uncharacterized protein LOC104118395 [*N. tomentosiformis*]
Niben101Scf00712g02011	0.04	0.38	0.17	0.04	0.08	39.41	41.88	121.75	192.66	5-*epi*-aristolochene synthase NbEAS7 [*N. benthamiana*]
Niben101Scf07201g01009	0.85	8.24	3.64	2.95	0.89	31.77	32.00	104.23	182.99	AAA-ATPase-like [*N. attenuata*]
Niben101Scf00577g09001	2.27	1.16	1.55	4.40	1.19	2.19	8.30	216.07	158.08	Hypothetical protein A4A49_37911 [*N. attenuata*]
Niben101Scf01534g02014	0.00	0.21	0.75	0.24	0.06	2.76	7.63	69.46	150.34	Zinc finger protein ZIC 2-like [*N. attenuata*]
Niben101Scf00821g16001	0.02	0.32	0.19	0.07	0.13	3.11	6.91	45.36	147.17	Somatic embryogenesis receptor kinase 2-like [*N. tabacum*]
Niben101Scf07491g00003	0.15	0.10	0.05	0.07	0.30	0.31	1.52	33.80	144.26	Endochitinase A [*N. tomentosiformis*]
Niben101Scf00313g07005	1.68	1.04	1.01	2.02	0.81	1.18	5.84	41.21	133.39	Benzyl alcohol O-benzoyltransferase [*N. attenuata*]
Niben101Scf08057g00006	0.20	1.07	0.67	1.02	1.03	12.03	17.16	78.40	127.41	Plant cadomiun resistance 2-like [*N. tabacum*]
Niben101Scf03374g06002	0.08	0.00	0.00	0.00	0.37	0.21	0.20	9.95	116.52	Lipid transfer-like protein VAS [*N. sylvestris*]
Niben101Scf05404g09001	0.04	0.50	0.50	0.30	0.25	2.83	4.46	21.65	115.96	Glutathione S-transferase [*N. sylvestris*]
Niben101Scf09044g01012	0.52	0.07	0.22	0.39	0.60	0.49	1.27	20.22	113.93	Osmotin [*N. sylvestris*]
Niben101Scf07767g02011	0.77	0.67	2.90	1.64	1.60	2.09	14.79	80.84	112.55	Cytochrome P450 71A1-like [*N. sylvestris*]
Niben101Scf01999g07002	0.07	0.10	0.11	0.58	0.74	0.44	1.68	24.87	112.37	Pathogenesis-related protein 1C-like [*N. sylvestris*]
Niben101Scf04787g02002	0.01	0.03	0.02	0.00	0.00	1.58	10.50	84.68	111.26	9-divinyl ether synthase [*N. tabacum*]
Niben101Scf00354g01012	0.02	0.00	0.00	0.00	0.02	0.05	2.27	34.86	109.31	deacetylvindoline O-acetyltransferase-like [*N. sylvestris*]
Niben101Scf00700g00005	0.01	1.27	0.24	0.06	0.03	41.47	29.76	73.28	105.38	5-*epi*-aristolochene synthase NbEAS6 [*N. benthamiana*]
Niben101Scf03993g06006	0.06	4.49	1.47	0.35	0.13	220.63	117.72	153.02	102.64	5-*epi*-aristolochene synthase NbEAS3 [*N. benthamiana*]
Niben101Scf12045g06025	0.54	0.84	1.81	1.67	0.95	3.03	4.61	7.59	101.52	Pathogenesis-related protein PR-4B [*N. tomentosiformis*]
Niben101Scf03096g01022	0.00	0.43	1.11	0.07	0.07	14.71	29.48	14.21	87.06	Cysteine-rich repeat secretory protein 38-like [*N. tabacum*]
Niben101Scf01942g04001	0.03	0.12	0.02	0.12	0.07	0.77	6.02	79.56	85.73	WRKY transcription factor 51 [*N. sylvestris*]
Niben101Scf01249g06013	0.01	1.31	0.35	0.17	0.04	5.95	7.20	59.02	85.59	Mitochondrial phosphate carrier protein 3 [*N. attenuata*]
Niben101Scf04944g05002	0.70	0.50	0.25	0.19	0.46	0.47	3.73	80.35	85.01	WRKY transcription factor 40 [*N. sylvestris*]
Niben101Scf01084g01009	0.17	0.41	0.19	0.45	0.26	3.47	9.87	34.06	84.57	NbSAR8.2b gene product [*N. benthamiana*]
Niben101Scf16114g01003	0.43	0.07	0.17	0.30	0.24	0.99	2.21	24.79	84.15	GDSL esterase/lipase 5-like [*N. tabacum*]
Niben101Scf06583g03008	0.47	1.40	0.53	0.54	0.24	2.75	5.78	63.57	83.64	PDR-type ACB transporter NbPDR2b [*N. benthamiana*]
Niben101Scf02410g00002	0.46	1.16	0.94	0.79	0.79	3.16	6.39	32.35	83.25	basic endochitinase [*N. attenuata*]
Niben101Scf06424g01007	0.07	0.28	0.26	0.06	0.19	0.49	0.47	5.93	80.44	Kunitz trypsin inhibitor 2-like [*N. attenuata*]

*Top 50 genes highly expressed in INF1-treated leaves 24 after the treatment.

Supplementary Table 7. *Nicotiana benthamiana* genes categorized in cluster 16.

Gene ID*	Expression (FPKM value)									Annotation (BlastX)
	0 h (Control)	H_2O				150 nM INF1				
		3 h	6 h	12 h	24 h	3 h	6 h	12 h	24 h	
Niben101Scf01239g02002	15.48	24.48	37.27	20.16	12.63	266.74	275.50	301.44	221.61	Caffeic acid 3-o-methyltransferase [*N. attenuata*]
Niben101Scf14679g00002	7.86	74.42	62.98	86.22	33.42	255.22	210.37	202.67	178.27	Secoisolariciresinol dehydrogenase-like [*N. attenuata*]
Niben101Scf07242g07006	11.32	42.33	23.98	24.63	11.52	119.78	105.29	174.13	160.51	Isoflavone 2'-hydroxylase-like [*N. sylvestris*]
Niben101Scf08127g08009	14.25	43.11	49.98	22.21	17.49	144.22	129.21	120.31	159.52	6-phosphogluconate dehydrogenase, decarboxylating 1 [*N. sylvestris*]
Niben101Scf02171g00007	0.44	8.28	8.73	2.60	2.79	119.65	104.75	112.09	156.17	Endochitinase PR4-like [*N. sylvestris*]
Niben101Scf00225g00008	6.67	10.53	20.79	8.99	4.64	173.25	211.25	242.64	142.15	Caffeic acid 3-o-methyltransferase [*N. attenuata*]
Niben101Scf02030g04003	14.33	43.93	84.07	15.93	15.11	131.06	171.13	71.67	128.26	Luminal-binding protein 4-like [*N. sylvestris*]
Niben101Scf01100g01006	11.62	19.92	14.66	13.42	11.42	92.14	68.73	128.61	125.41	acetyl-CoA acetyltransferase NbACAT1b [*N. benthamiana*]
Niben101Scf04083g04040	13.65	64.13	71.19	15.14	13.88	195.78	122.63	92.00	122.28	Luminal-binding protein 5 precursor [*N. tabacum*]
Niben101Scf10688g01013	3.56	20.07	7.06	8.25	4.25	57.26	54.07	141.83	120.20	Blue copper protein-like [*N. attenuata*]
Niben101Scf08590g00005	19.87	56.62	85.81	21.01	17.67	179.18	139.38	89.06	114.01	Luminal-binding protein 5 precursor [*N. tabacum*]
Niben101Scf08196g01001	8.12	91.99	88.23	29.97	6.15	394.72	363.31	83.10	112.11	Glutathione S-transferase parA [*N. sylvestris*]
Niben101Scf04563g01016	6.94	39.80	8.93	7.63	8.36	208.40	31.85	65.94	102.61	Methyltransferase DDB_G0268948 [*N. tabacum*]
Niben101Scf00031g02001	4.49	31.11	17.22	7.03	5.77	199.46	169.58	139.04	101.99	Epidermis-specific secreted glycoprotein EP1-like precursor [*N. tabacum*]
Niben101Scf22099g00006	3.42	23.84	10.41	4.18	5.02	65.97	70.73	90.85	91.44	Cytochrome b561 and DOMON domain-containing protein [*N. attenuata*]
Niben101Scf14394g01020	10.40	40.39	55.62	14.56	13.58	125.73	98.74	41.34	90.46	Luminal-binding protein [*N. sylvestris*]
Niben101Scf01729g01015	2.16	6.73	5.01	3.72	2.55	92.02	62.07	120.93	81.58	3-hydroxy-3-methylglutaryl-CoA synthase NbHMGS1b [*N. benthamiana*]
Niben101Scf00779g06009	9.06	70.58	22.10	13.65	8.46	193.65	60.42	68.57	81.37	12-oxophytodienoate reductase 2-like [*N. attenuata*]
Niben101Scf00372g05012	7.71	32.83	32.90	21.03	4.27	134.56	169.22	114.88	79.78	Hypothetical protein A4A49_08095 [*N. attenuata*]
Niben101Scf14069g00002	5.78	83.18	44.21	13.01	3.72	126.80	70.81	53.78	77.97	Cysteine-rich and transmembrane domain-containing protein A [*N. attenuata*]
Niben101Scf01719g08001	8.86	17.34	14.04	13.43	6.68	155.73	97.70	173.31	74.20	PDR-type ACB transporter NbPDR1a [*N. benthamiana*]
Niben101Scf03438g02001	0.03	24.02	4.85	5.91	1.59	29.70	19.85	25.66	73.02	Heat stress transcription factor B-3-like [*N. tabacum*]
Niben101Scf01596g12004	8.90	41.68	49.57	11.28	9.45	110.67	83.66	45.19	71.86	Calnexin homolog 1-like [*N. sylvestris*]
Niben101Scf02907g06031	4.83	48.97	17.50	6.15	5.49	253.91	82.05	85.73	69.25	Mannitol dehydrogenase [*N. tomentosiformis*]
Niben101Scf00173g05015	9.28	9.69	9.67	9.71	9.85	48.83	48.67	76.54	69.04	Mevalonate-5-pyrophosphate decarboxylase NbMVD1b [*N. benthamiana*]
Niben101Scf02353g06039	0.84	14.62	12.57	6.24	4.57	77.78	70.00	57.84	65.98	Kunitz trypsin inhibitor 2-like [*N. attenuata*]
Niben101Scf01241g02004	1.19	37.27	29.24	11.71	5.15	182.55	85.53	42.96	64.76	Glutamate decarboxylase 4 [*N. tabacum*]
Niben101Scf07395g00029	10.75	35.72	36.11	20.32	12.80	150.39	71.41	62.27	63.51	NRT1/PTR family 2.11-like [*N. tabacum*]
Niben101Scf04003g08001	5.54	11.79	6.94	5.34	5.42	57.96	98.26	131.20	62.16	Caffeoyl-CoA O-methyltransferase 4 [*N. tabacum*]
Niben101Scf12738g00010	4.30	37.50	28.66	34.61	10.36	114.92	114.47	35.09	59.37	Ferredoxin, root R-B2-like [*N. tomentosiformis*]
Niben101Scf09203g01012	6.75	10.90	31.05	6.78	6.87	53.81	70.40	38.49	57.78	GTP-binding protein SAR2 [*N. tomentosiformis*]
Niben101Scf00031g01005	3.89	48.25	22.22	5.33	5.43	196.70	145.56	104.91	52.58	Epidermis-specific secreted glycoprotein EP1-like [*N. tabacum*]
Niben101Scf04122g05014	6.07	17.43	4.74	2.65	2.68	34.44	17.00	43.74	52.09	Sugar carrier protein C-like [*N. sylvestris*]
Niben101Scf00637g04001	10.19	67.10	42.47	32.03	13.12	260.39	219.03	112.55	51.33	Polygalacturonase inhibitor-like [*N. attenuata*]
Niben101Scf03115g02008	5.17	26.45	31.24	5.74	5.14	84.14	53.32	32.52	51.30	Luminal-binding protein 4 [*N. attenuata*]
Niben101Scf06394g00009	7.98	37.22	22.93	12.84	7.89	144.51	33.78	29.95	51.16	2-hydroxyacyl-CoA lyase [*N. attenuata*]
Niben101Scf06779g01002	3.52	5.55	11.40	3.64	3.81	36.54	35.96	28.46	51.04	Inorganic phosphate transporter 1-4-like [*N. sylvestris*]
Niben101Scf00773g08003	1.97	5.80	3.74	1.79	2.29	47.79	51.32	55.86	50.33	Wound-induced protein 1-like, partial [*N. sylvestris*]
Niben101Scf01789g03003	3.67	15.84	20.39	3.90	5.93	107.06	52.29	16.89	48.32	Chitinase-3-like protein 1 [*N. sylvestris*]
Niben101Scf13180g01003	1.43	14.03	2.66	1.91	2.24	16.59	34.19	45.97	46.94	3-hydroxy-3-methylglutaryl-coenzyme A reductase NbHMGR1a [*N. benthamiana*]
Niben101Scf07451g00013	2.07	10.68	4.00	2.70	3.30	22.48	41.61	42.19	45.40	Calcium-binding allergen Ole e 8-like [*N. tabacum*]
Niben101Scf03413g01011	11.91	35.44	25.78	16.18	11.32	135.13	74.37	58.95	44.34	Mevalonate-5-pyrophosphate decarboxylase NbMVD1a [*N. benthamiana*]
Niben101Scf06009g00025	3.16	7.61	4.52	4.45	2.95	38.90	27.04	51.71	43.58	SNAP25 homologous protein SNAP33 [*N. sylvestris*]
Niben101Scf01432g06023	3.81	8.60	4.55	3.52	3.08	29.46	88.79	82.72	43.45	Caffeoyl-CoA O-methyltransferase 3 [*N. tabacum*]
Niben101Scf09030g01004	2.92	12.33	7.89	5.16	5.39	45.88	56.24	61.17	41.95	Serine/arginine repetitive matrix protein 2-like [*N. sylvestris*]
Niben101Scf03710g10004	7.50	26.90	27.09	18.76	16.51	107.76	80.15	48.87	41.45	Amino acid permease 3-like [*N. sylvestris*]
Niben101Scf06583g03009	1.24	28.38	5.84	4.09	1.65	162.75	59.45	80.33	40.47	PDR-type ACB transporter NbPDR1b [*N. benthamiana*]
Niben101Scf00372g05013	5.08	28.84	43.72	14.18	2.58	155.11	189.61	98.97	39.84	Hypothetical protein A4A49_08095 [*N. attenuata*]
Niben101Scf07586g00004	0.84	8.13	3.20	2.35	1.77	27.18	31.39	40.01	39.27	Calcium-binding protein CAST-like [*N. sylvestris*]
Niben101Scf25768g00010	4.68	8.42	8.50	6.68	3.08	30.86	17.46	34.61	39.09	Receptor-like protein kinase FERONIA [*N. tabacum*]

*Top 50 genes highly expressed in INF1-treated leaves 24 after the treatment.

Supplementary Table 8. Predicted *Nicotiana benthamiana* genes for enzymes specifically involved in the production of capsidiol.

Gene Name	Gene ID	Cluster ID[1]
**5-*epi*-Aristolochene synthase (EAS)**		
NbEAS1	Niben101Scf07725g01004	**14**
NbEAS2	Niben101Scf07725g00004	**14**
NbEAS3	Niben101Scf03993g06006	**14**
NbEAS4	Niben101Scf03993g05005	**14**
NbEAS5	Niben101Scf06245g01017	**14**
NbEAS6	Niben101Scf00700g00005	**14**
NbEAS7	Niben101Scf00712g02011	**14**
NbEAS8	Niben101Scf04362g07009	**14**
NbEAS9	Niben101Scf01683g03005	**14**
NbEAS10	Niben101Scf03400g02010	**14**
**5-*epi*-Aristolochene dihydroxylase (EAH)**		
NbEAH1	Niben101Scf00072g06001	**14**
NbEAH2	Niben101Scf04362g07011	**14**
NbEAH3	Niben101Scf00072g05003	**14**
NbEAH4	Niben101Scf00072g05004	**14**
NbEAH5	Niben101Scf00994g00001	**14**
NbEAH6	Niben101Scf04869g00002	n/a

[1] Cluster numbers for INF1-induced genes are shown in bold.
n/a, not assigned.

Supplementary Table 9. Predicted genes for enzymes in mevalonate pathway and farnesylpyrophosphate synthase in *Nicotiana benthamiana.*

Gene Name	Gene ID	**Cluster ID**[1]
**Acetyl-CoA thiolase (ACAT)**		
NbACAT1a[2]	Niben101Scf04727g03006	3
NbACAT1b	Niben101Scf01100g01006	**16**
NbACAT2a	Niben101Scf06423g02006	8
NbACAT2b	Niben101Scf01974g00019	7
NbACAT3	Niben101Scf05433g00004	9
**3-Hydroxy-3-methylglutaryl-CoA synthase (HMGS)**		
NbHMGS1a[2]	Niben101Scf01111g01003	**4**
NbHMGS1b	Niben101Scf01729g01015	**16**
NbHMGS2a	Niben101Scf03321g01012	9
NbHMGS2b	Niben101Scf02361g01002	2
NbHMGS3	Niben101Scf10595g01007	13
**3-Hydroxy-3-methylglutaryl-CoA reductase (HMGR)**		
NbHMGR1a	Niben101Scf13180g01003	**16**
NbHMGR1b	Niben101Scf09686g00013	**2**
NbHMGR2[2]	Niben101Scf02203g05002	**14**
NbHMGR3a	Niben101Scf09883g01009	13
NbHMGR3b	Niben101Scf00163g01009	1
**Mevalonate-5-kinase (MVK)**		
NbMVK1a	Niben101Scf25893g00005	**2**
NbMVK1b	Niben101Scf00370g03023	**4**
**Phosphomevalonate kinase (PMVK)**		
NbPMVK1a	Niben101Scf07030g04004	**2**
NbPMVK1b	Niben101Scf09628g00020	8
**Mevalonate-5-pyrophosphate decarboxylase (MVD)**		
NbMVD1a[2]	Niben101Scf03413g01011	**16**
NbMVD1b	Niben101Scf00173g05015	**16**
**Isopentenyl pyrophosphate isomerase (IPPS)**		
NbIPPI1a	Niben101Scf17839g02005	**2**
NbIPPI1b	Niben101Scf05848g05012	**4**
NbIPPI2a	Niben101Scf02499g03007	**2**
NbIPPI2b	Niben101Scf01514g04018	9
**Farnesylpyrophosphate synthase (FPPS)**		
NbFPPS1a[2]	Niben101Scf04739g01006	**16**
NbFPPS1b	Niben101Scf00414g07005	**4**
NbFPPS2a	Niben101Scf04847g02011	7
NbFPPS2b	Niben101Scf04444g09012	**4**

[1] Cluster numbers for INF1-induced genes are shown in bold.

[2] Identified as an essential gene for the resistance to *P. infestans* in Shibata et al., 2016.

Supplementary Table 10. Predicted genes for enzymes involved in methionine cycle and ethylene production in *Nicotiana benthamiana* .

Gene Name	Gene ID	Cluster ID[1]
**Cystathionine gamma-synthase**		
NbCGS1a [2]	Niben101Scf10866g01001	**2**
NbCGS1b	Niben101Scf00136g03005	9
NbCGS2a	Niben101Scf04133g01027	13
NbCGS2b	Niben101Scf06457g00005	13
**Cystathionine beta-lyase**		
NbCBL1a	Niben101Scf08333g07030	9
NbCBL1b	Niben101Scf03006g06008	9
NbCBL2 [3]	Niben101Scf05535g03002	7
**Methionine synthase**		
NbMS1a	Niben101Scf09725g00022	1
NbMS1b	Niben101Scf01812g02025	1
NbMS2	Niben101Scf07438g04014	13
NbMS3	Niben101Scf03634g04009	13
NbMS4a	Niben101Scf00054g01013	13
NbMS4b	Niben101Scf12280g00002	13
NbMS5a	Niben101Scf09813g00005	8
NbMS5b	Niben101Scf05629g01024	8
NbMS6 [3]	Niben101Scf10678g00010	8
**S-adenosylmethionine (SAM) synthetase**		
NbSAMS1a [2, 4]	Niben101Scf03535g01001	1
NbSAMS1b	Niben101Scf04643g02010	11
NbSAMS2a [5]	Niben101Scf11751g01007	3
NbSAMS2b	Niben101Scf02502g04001	3
NbSAMS3a	Niben101Scf01236g02016	13
NbSAMS3b	Niben101Scf01820g00025	13
NbSAMS4a	Niben101Scf01861g00002	**2**
NbSAMS4b	Niben101Scf00402g04010	9
NbSAMS5	Niben101Scf00285g00001	13
NbSAMS6	Niben101Scf01334g06003	7
NbSAMS7a	Niben101Scf15054g01008	17
NbSAMS7b	Niben101Scf04473g14015	n. a.
NbSAMS8	Niben101Scf01671g03004	12
NbSAMS9 [3]	Niben101Ctg06829g00002	13
NbSAMS10 [3]	Niben101Scf00402g04005	9
**S-adenosylhomocycteine (SAH) hydrolase**		
NbSAHH1a [2, 6]	Niben101Scf10608g02009	1
NbSAHH1b	Niben101Scf09136g00004	1
NbSAHH2a	Niben101Scf01171g03016	7
NbSAHH2b	Niben101Scf01592g01001	13
NbSAHH3	Niben101Scf06737g00025	5
NbSAHH [3]	Niben101Scf02853g05019<br>Niben101Scf02853g05018	5
**Aminocyclopropane carboxylate (ACC) synthase**		
NbACS1	Niben101Scf09512g03008	**14**
NbACS2a	Niben101Scf06180g00015	**2**

NbACS2b	Niben101Scf02334g00004	11
NbACS3	Niben101Scf00254g00004	8
NbACS4	Niben101Scf03226g01003	**2**
NbACS5a	Niben101Scf05348g01023	19
NbACS5b	Niben101Scf05977g01005	19
NbACS6a	Niben101Scf03907g01018	13
NbACS6b	Niben101Scf00485g02022	8
NbACS7a	Niben101Scf00388g07005	20
NbACS7b	Niben101Scf15817g01015	20
NbACS8a	Niben101Scf02740g15001	n. a.
NbACS8b	Niben101Scf11516g00011	n. a.
NbACS9a	Niben101Scf04773g00021	n. a.
NbACS9b	Niben101Scf02081g03012	n. a.
NbACS10a	Niben101Scf02053g00003	n. a.
NbACS10b	Niben101Ctg13861g00002	n. a.
NbACS11a	Niben101Scf05449g01004	n. a.
NbACS11b	Niben101Scf00108g05008	n. a.
NbACS12a	Niben101Scf00926g03006	n. a.
NbACS12b	Niben101Scf08472g00003	n. a.
NbACS13a	Niben101Scf03238g02003	n. a.
NbACS13b	Niben101Scf02816g05004	n. a.
NbACS14	Niben101Scf39461g00001	n. a.
**Aminocyclopropane carboxylate (ACC) oxydase**		
NbACO1a [2]	Niben101Scf02543g01008	**10**
NbACO1b [2]	Niben101Scf08039g01005	**10**
NbACO2a [2]	Niben101Scf09590g03007 (LC008355)[7]	3
NbACO2b	Niben101Scf02433g06003 (LC659235)[7]	9
NbACO3a	Niben101Scf09217g00024	19
NbACO3b	Niben101Scf13622g01020	9
NbACO4	Niben101Scf02918g00003	**14**
NbACO5a	Niben101Scf19336g00004	n. a.
NbACO5b	Niben101Scf00313g01001	n. a.
NbACO6	Niben101Scf00313g01004	n. a.
NbACO7a	Niben101Scf02597g08002	19
NbACO7b	Niben101Scf00430g00002	n. a.
NbACO8a	Niben101Scf14041g00001	1
NbACO8b	Niben101Scf03710g07016	19
NbACO9a	Niben101Scf09834g00001	8
NbACO9b	Niben101Scf15670g00014	**4**

[1] Cluster numbers for INF1-induced genes are shown in bold.

[2] Genes essential for the resistance to *P. infestans* (Shibata et al. 2016)

[3] Probable pseudogene or non-coding sequence.

[4] Most homologous to *NbSAM1* in Ismayil et al. (2018).

[5] Most homologous to *NbSAM2* and *NbSAM3* in Ismayil et al. (2018).

[6] Reported as *NbSAHH* in Carmen Cañizares et al. (2013).

[7] The sequences with the Niben101 ID numbers are partial sequences. Full-length cDNA sequences were constructed by *de novo* assembly of RNAseq data and registered unde[illegible] accession nos. shown in parentheses.

n/a, not assigned.

Supplementary Table 11. Gene list for predicted AP2/RAV family transcription factors in *Nicotiana benthamiana* .

Family	Gene ID	Gene Name	Arabidopsis homologues in same branch of phylogenetic tree		Cluster
AP2	Niben101Scf07223g00002	*NbAP2-1*			12
	Niben101Scf07050g00010	*NbAP2-2a*			13
	Niben101Scf00496g02009	*NbAP2-2b*			8
	Niben101Scf07944g03004	*NbAP2-3a*	AP2	At4g36920.1	13
	Niben101Scf01692g01003	*NbAP2-3b*	TOE3	At5g67180.1	8
	Niben101Scf01432g01005	*NbAP2-4a*			7
	Niben101Scf07444g03006	*NbAP2-4b*			13
	Niben101Scf00280g01007	*NbAP2-5a*			13
	Niben101Scf25996g00006	*NbAP2-5b*			13
	Niben101Scf01035g04005	*NbAP2-6a*			1
	Niben101Scf02702g00005	*NbAP2-6b*	TOE1/RAP2.7	At2g28550.3	8
	Niben101Scf11756g01019	*NbAP2-7a*	TOE2	At5g60120.2	7
	Niben101Scf03883g05010	*NbAP2-7b*	SNZ	At2g39250.1	8
	Niben101Scf02369g04004	*NbAP2-8a*	SMZ	At3g54990.1	11
	Niben101Scf00482g08004	*NbAP2-8b*			18
	Niben101Scf00396g00013	*NbAP2-9*			19
	Niben101Scf07196g01001	*NbAP2-10*	PLT1	At3g20840.1	n. a.
	Niben101Scf07196g01008	*NbAP2-11a*	PLT2	At1g51190.1	9
	Niben101Scf07196g01007	*NbAP2-11b*			9
	Niben101Scf04386g00002	*NbAP2-12*			n. a.
	Niben101Scf11138g01008	*NbAP2-13a*	-	At2g41710.3	8
	Niben101Scf02156g03004	*NbAP2-13b*			9
	Niben101Scf07030g00017	*NbAP2-14a*			13
	Niben101Scf15227g00026	*NbAP2-14b*			1
	Niben101Scf03437g02045	*NbAP2-15a*	WRI3	At1g16060.1	13
	Niben101Scf04218g00013	*NbAP2-15b*	WRI4	At1g79700.2	9
	Niben101Scf00546g00002	*NbAP2-16*			7
	Niben101Scf05109g04005	*NbAP2-17a*			n. a.
	Niben101Scf08876g00008	*NbAP2-17b*			n. a.
	Niben101Scf02891g04012	*NbAP2-18a*	WRI1	At3g54320.1	7
	Niben101Scf13694g00020	*NbAP2-18b*			8
	Niben101Scf01774g10021	*NbAP2-19a*			9
	Niben101Scf03419g03008	*NbAP2-19b*	ANT	At4g37750.1	8
	Niben101Scf00388g03006	*NbAP2-20a*			7
	Niben101Scf07169g02008	*NbAP2-20b*			9
	Niben101Scf11515g00016	*NbAP2-21a*			12
	Niben101Scf07511g02006	*NbAP2-21b*	AIL6	At5g10510.3	n. a.
	Niben101Scf04574g07001	*NbAP2-22a*	AIL7	At5g65510.1	1
	Niben101Scf00173g09005	*NbAP2-22b*			n. a.
	Niben101Scf01438g04007	*NbAP2-23a*	AIL5	At5g57390.1	13
	Niben101Scf02540g00013	*NbAP2-23b*			7
	Niben101Scf04691g00002	*NbAP2-24a*	BBM	At5g17430.1	n. a.
	Niben101Scf06407g01001	*NbAP2-24b*			n. a.
	Niben101Scf05123g00001	*NbAP2-25a*	AIL1	At1g72570.1	11
	Niben101Scf02749g05039	*NbAP2-25b*			1
Soloist I	Niben101Scf03479g03001	*NbAP2-S-1a*	APD1	At4g13040.3	8
	Niben101Scf00113g03002	*NbAP2-S-1b*			**2**
RAV	Niben101Scf10767g03010	*NbRAV-1*	RAV2/RAP2.8	At1g68840.1	8
	Niben101Scf06059g01001	*NbRAV-2a*	EDF3	At3g25730.1	11
	Niben101Scf16806g00003	*NbRAV-2b*	TEM1	At1g25560.1	n. a.
	Niben101Scf03150g06002	*NbRAV-3a*	RAV1/EDF4	At1g13260.1	**16**
	Niben101Scf00872g03005	*NbRAV-3b*			11
	Niben101Scf07498g01011	*NbRAV-4a*	-	At1g50680.1	n. a.
	Niben101Scf03488g06004	*NbRAV-4b*	-	At1g51120.1	n. a.

[1] Cluster numbers for INF1-induced genes are shown in bold.
n. a., not assigned.

Supplementary Table 12. Gene list for predicted ERF family transcription factors in *Nicotiana benthamiana*.

Sub family		Gene ID	Gene Name	Arabidopsis homologues in same branch of phylogenetic tree		Cluster[1]
ERF-I, -IIa	(DREB A-6)	Niben101Scf01177g01001	*NbERF-I-1a*			18
		Niben101Scf09459g02008	*NbERF-I-1b*	ERF15	At4g31060.1	6
		Niben101Scf13695g01014	*NbERF-I-2a*	ERF61	At1g64380.1	13
		Niben101Scf18347g00023	*NbERF-I-2b*	ERF62	At4g13620.1	13
		Niben101Scf03850g01005	*NbERF-I-3a*			11
		Niben101Scf02972g00008	*NbERF-I-3b*			3
		Niben101Scf06119g02002	*NbERF-I-4a*	TG (ERF55)	At1g36060.1	8
		Niben101Scf01980g04003	*NbERF-I-4b*	ERF56	At2g22200.1	11
		Niben101Scf02219g02002	*NbERF-I-5a*	WIND4 (ERF57)	At5g65130.1	7
		Niben101Scf10283g01004	*NbERF-I-5b*	RAP2.4D/WIND2 (ERF58)	At1g22190.1	13
		Niben101Scf03510g06011	*NbERF-I-6a*	RAP2.4/WIND1 (ERF59)	At1g78080.1	8
		Niben101Scf00846g02001	NbERF-I-6b	ERF60	At4g39780.1	8
		Niben101Scf05440g02002	*NbERF-I-7a*			9
		Niben101Scf34675g00005	*NbERF-I-7b*	ERF53	At2g20880.1	n. a.
		Niben101Scf06117g01012	*NbERF-I-8a*	ERF54	At4g28140.1	13
		Niben101Scf06120g00005	*NbERF-I-8b*			3
		Niben101Scf10763g00021	*NbERF-I-9*			n.a.
	(DREB A-5)	Niben101Scf01692g03007	*NbERF-II-1a*			8
		Niben101Scf04826g01005	*NbERF-II-1b*	RAP2.1 (ERF6)	At1g46768.1	13
		Niben101Scf07066g03005	*NbERF-II-2a*	RAP2.9 (ERF7)	At4g06746.1	9
		Niben101Scf05135g06001	*NbERF-II-2b*	DEAR3 (ERF8)	At2g23340.1	3
		Niben101Scf06444g02001	*NbERF-II-3a*	RAP2.10 (ERF9)	At4g36900.1	7
		Niben101Scf03314g00004	*NbERF-II-3b*	DEAR2 (ERF10)	At5g67190.1	1
		Niben101Scf03223g00002	*NbERF-II-4*	CEJ1 (ERF11)	At3g50260.1	19
ERF-IIb, -IIc	(DREB A-5)	Niben101Scf15963g01004	*NbERF-II-5a*	ERF19	At1g22810.1	n. a.
		Niben101Scf06473g01014	*NbERF-II-5b*	ERF20	At1g71520.1	n. a.
		Niben101Scf06596g03021	*NbERF-II-6a*	DREB26 (ERF12)	At1g21910.1	1
		Niben101Scf09087g00009	*NbERF-II-6b*	ERF13	At1g77640.1	12
				ERF14	At1g44830.1	1
		Niben101Scf00298g00001	*NbERF-II-7a*			n. a.
		Niben101Scf02437g08005	*NbERF-II-7b*	ERF16	At5g21960.1	17
		Niben101Scf11694g01005	*NbERF-II-8*	ERF17	At1g19210.1	n. a.
		Niben101Scf05773g00002	*NbERF-II-9*	ORA47 (ERF18)	At1g74930.1	n. a.
		Niben101Scf02744g00004	*NbERF-II-10a*			n. a.
		Niben101Scf08651g04017	*NbERF-II-10b*			n. a.
ERF-III	(DREB A-4)	Niben101Scf01611g11002	*NbERF-III-1a*			13
		Niben101Scf05156g01011	*NbERF-III-1b*	ERF43	At4g32800.1	13
		Niben101Scf08776g00003	*NbERF-III-2*	ESE2 (ERF42)	At2g25820.1	n. a.
		Niben101Scf09363g00010	*NbERF-III-3a*	ERF37	At1g77200.1	n. a.
		Niben101Scf01374g05001	*NbERF-III-3b*	TINY (ERF40)	At5g25810.1	**2**
		Niben101Scf01539g00001	*NbERF-III-4*	TINY2 (ERF41)	At5g11590.1	11
		Niben101Scf06969g00003	*NbERF-III-5*	ERF23	At1g01250.1	**2**
		Niben101Scf04337g01005	*NbERF-III-6*	ERF36	At3g16280.1	13
		Niben101Scf00603g02001	*NbERF-III-7*			13
		Niben101Scf05855g06014	*NbERF-III-8a*			11
		Niben101Scf00315g02001	*NbERF-III-8b*	ERF34	At2g44940.1	n. a.
		Niben101Scf07693g00011	*NbERF-III-9a*	ERF35	At3g60490.1	6
		Niben101Scf06436g05001	*NbERF-III-9b*	ERF38	At2g35700.1	3
		Niben101Scf15704g00006	*NbERF-III-10*	ERF39	At4g16750.1	13
		Niben101Scf06157g01005	*NbERF-III-11a*			11
		Niben101Scf03772g00001	*NbERF-III-11b*			n. a.
	(DREB A-1)	Niben101Scf03321g01009	*NbERF-III-12a*			20
		Niben101Scf03092g00007	*NbERF-III-12b*			n. a.
		Niben101Scf05459g00001	*NbERF-III-13*			n. a.
		Niben101Ctg16385g00003	*NbERF-III-14*		At1g12610.1	n. a.
		Niben101Scf03245g04004	*NbERF-III-15a*	DDF1 (ERF33)	At1g63030.1	n. a.
		Niben101Scf03245g04005	*NbERF-III-15b*	DDF2 (ERF32)	At4g25490.1	n. a.
		Niben101Scf16007g00010	*NbERF-III-16a*	CBF1/DREB1B (ERF29)	At4g25470.1	n. a.
		Niben101Scf16007g00011	*NbERF-III-16b*	CBF2/DREB1C (ERF30)	At4g25480.1	n. a.
		Niben101Scf00282g02006	*NbERF-III-17*	CBF3/DREB1A (ERF31)	At5g51990.1	3
		Niben101Scf00085g00008	*NbERF-III-18a*			n. a.
		Niben101Scf06081g02026	*NbERF-III-18b*			n. a.
	(DREB A-4)	Niben101Scf04217g07002	*NbERF-III-19*			n. a.
		Niben101Scf08840g00003	*NbERF-III-20a*			6
		Niben101Scf08840g00008	*NbERF-III-20b*			**16**
		Niben101Scf16506g00004	*NbERF-III-21*	HRD (ERF24)	At2g36450.1	n. a.
		Niben101Scf01111g02001	*NbERF-III-22*	ERF25	At5g52020.1	n. a.
		Niben101Scf03245g06003	*NbERF-III-23a*	ERF26	At1g63040.1	11
		Niben101Scf02577g03014	*NbERF-III-23b*	ERF27	At1g12630.1	n. a.
		Niben101Scf00784g01003	*NbERF-III-24*			n. a.

		Niben101Scf03580g01003	*NbERF-III-25a*			n. a.
		Niben101Scf11954g01002	*NbERF-III-25b*			n. a.
		Niben101Scf06848g08006	*NbERF-III-26a*			n. a.
		Niben101Scf02182g19004	*NbERF-III-26b*	FUF1 (ERF21)	At1g71450.1	n. a.
		Niben101Scf06848g09002	*NbERF-III-27a*	ERF22	At1g33760.1	n. a.
		Niben101Scf02182g21007	*NbERF-III-27b*			n. a.
ERF-IV	(DREB-A2)	Niben101Scf00735g02005	*NbERF-IV-1a*			1
		Niben101Scf05993g01013	*NbERF-IV-1b*	DREB2F (ERF51)	At3g57600.1	12
		Niben101Scf04505g02007	*NbERF-IV-2*			15
		Niben101Scf00177g02016	*NbERF-IV-3*	DREB2G (ERF50)	At5g18450.1	11
		Niben101Scf01701g03007	*NbERF-IV-4a*			n. a.
		Niben101Scf08033g01007	*NbERF-IV-4b*	DREB2D (ERF49)	At1g75490.1	n. a.
		Niben101Scf07226g08005	*NbERF-IV-5*			11
		Niben101Scf10169g00004	*NbERF-IV-6a*			8
		Niben101Scf01237g06003	*NbERF-IV-6b*	DREB2B (ERF44)	At3g11020.1	**10**
		Niben101Scf06378g04004	*NbERF-IV-7a*	DREB2A (ERF45)	At5g05410.1	8
		Niben101Scf08060g00005	*NbERF-IV-7b*	DREB19 (ERF46)	At2g38340.1	8
		Niben101Scf03202g13015	*NbERF-IV-8a*	DREB2H (ERF47)	At2g40350.1	n. a.
		Niben101Scf01297g01001	*NbERF-IV-8b*	DREB2C (ERF48)	At2g40340.1	n. a.
		Niben101Scf05849g00002	*NbERF-IV-9*			**2**
ERF-V		Niben101Scf04131g01028	*NbERF-V-1a*			17
		Niben101Scf04131g01007	*NbERF-V-1b*			n. a.
		Niben101Scf05800g00005	*NbERF-V-2*			n. a.
		Niben101Scf07926g07004	*NbERF-V-3a*	ESE3 (ERF3)	At5g25190.1	7
		Niben101Scf04437g00010	*NbERF-V-3b*			17
		Niben101Scf05618g01002	*NbERF-V-4*			n. a.
		Niben101Scf01142g04009	*NbERF-V-5a*			8
		Niben101Scf06190g06004	*NbERF-V-5b*			3
		Niben101Scf02397g00007	*NbERF-V-6a*			n. a.
		Niben101Scf09659g03018	*NbERF-V-6b*			n. a.
		Niben101Scf01669g04022	*NbERF-V-7*			n. a.
		Niben101Scf02475g08017	*NbERF-V-8a*			n. a.
		Niben101Scf03732g03003	*NbERF-V-8b*			n. a.
		Niben101Scf04763g00013	*NbERF-V-9a*			1
		Niben101Scf04763g00014	*NbERF-V-9b*	RAP2.11 (ERF2)	At5g19790.1	13
		Niben101Scf06026g00002	*NbERF-V-10*			**2**
		Niben101Scf17051g00007	*NbERF-V-11*			n. a.
		Niben101Scf15962g00025	*NbERF-V-12a*			n. a.
		Niben101Scf06348g00034	*NbERF-V-12b*			n. a.
		Niben101Scf11303g01019	*NbERF-V-13a*			8
		Niben101Scf11341g00009	*NbERF-V-13b*			**16**
		Niben101Scf09882g01001	*NbERF-V-14a*			5
		Niben101Ctg12291g00001	*NbERF-V-14b*	SHN1/WIN1 (ERF1)	At1g15360	n. a.
		Niben101Scf03930g01015	*NbERF-V-15*	SHN2 (ERF4)	At5g11190.1	n. a.
		Niben101Scf07913g00005	*NbERF-V-16*	SHN3 (ERF5)	At5g25390.2	15
		Niben101Scf12609g01006	*NbERF-V-17a*			11
		Niben101Scf03832g02022	*NbERF-V-17b*			11
ERF-VI		Niben101Ctg15705g00001	*NbERF-VI-1a*			13
		Niben101Ctg15705g00004	*NbERF-VI-1b*			13
		Niben101Scf00725g02012	*NbERF-VI-2*			13
		Niben101Scf01075g01003	*NbERF-VI-3*			19
		Niben101Scf00753g02005	*NbERF-VI-4*	CRF9 (ERF117)	At1g49120.1	9
		Niben101Scf04409g00010	*NbERF-VI-5a*			11
		Niben101Scf03817g12030	*NbERF-VI-5b*			7
		Niben101Scf08575g01003	*NbERF-VI-6a*			13
		Niben101Scf02222g00019	*NbERF-VI-6b*			17
		Niben101Scf00278g05001	*NbERF-VI-7a*			11
		Niben101Scf00530g01001	*NbERF-VI-7b*			8
		Niben101Scf08278g00007	*NbERF-VI-8*			7
		Niben101Scf02726g03006	*NbERF-VI-9a*	CRF1 (ERF63)	At4g11140.1	1
		Niben101Scf01543g03001	*NbERF-VI-9b*	CRF2/TMO3 (ERF64)	At4g23750.1	13
		Niben101Scf00503g07005	*NbERF-VI-10a*	CRF3 (ERF65)	At5g53290.1	**4**
		Niben101Scf03773g00009	*NbERF-VI-10b*	CRF4 (ERF66)	At4g27950.1	**4**
		Niben101Ctg13994g00001	*NbERF-VI-11a*	CRF6 (ERF67)	At3g61630.1	19
		Niben101Scf01817g01001	*NbERF-VI-11b*	CRF5 (ERF68)	At2g46310.1	19
		Niben101Scf03985g01005	*NbERF-VI-12*	CRF7 (ERF69)	At1g22985.1	13
		Niben101Scf01001g06011	*NbERF-VI-13a*	CRF8 (ERF70)	At1g71130.1	9
		Niben101Scf09004g00001	*NbERF-VI-13b*			9
		Niben101Scf09004g00003	*NbERF-VI-14*			n. a.
		Niben101Scf01518g08004	*NbERF-VI-15a*			8
		Niben101Scf00577g11004	*NbERF-VI-15b*			9
		Niben101Scf00262g04006	*NbERF-VI-16a*			3
		Niben101Scf02316g03001	*NbERF-VI-16b*	CRF10 (ERF118)	At1g68550.1	3
		Niben101Scf04860g02011	*NbERF-VI-17*	CRF11 (ERF119)	At3g25890	n. a.
		Niben101Scf01695g01005	*NbERF-VI-18*	CRF12 (ERF116)	At1g25470.1	8

	Niben101Scf12874g00001	*NbERF-VI-19*			n. a.
	Niben101Scf08196g00012	*NbERF-VI-20a*			**4**
	Niben101Scf01085g03003	*NbERF-VI-20b*			**4**
ERF-VII	Niben101Scf06913g00001	*NbERF-VII-1a*			3
	Niben101Scf01956g05006	*NbERF-VII-1b*			**2**
	Niben101Scf01430g00007	*NbERF173*[2]*/NbERF-VII-2*	ERF71	At2g47520.1	3
	Niben101Scf05827g07015	*NbERF-VII-3a*	AtEBP/RAP2.3 (ERF72)	At3g16770.1	9
	Niben101Scf11139g00001	*NbERF-VII-3b*	ERF73	At1g72360.1	9
	Niben101Scf19733g00001	*NbERF-VII-4a*	RAP2.12 (ERF74)	At1g53910.1	3
	Niben101Scf05123g02008	*NbERF-VII-4b*	RAP2.2 (ERF75)	At3g14230.1	7
	Niben101Scf00870g05003	*NbERF-VII-5*			7
ERF-VIII	-	-	ERF112	At2g33710.2	n. a.
			ESR1 (ERF89)	At1g12980.1	n. a.
	Niben101Scf01818g07006	*NbERF-VIII-1a*			n. a.
	Niben101Scf10785g01002	*NbERF-VIII-1b*			n. a.
	Niben101Scf10785g01014	*NbERF-VIII-2*			n. a.
	Niben101Scf01818g08019	*NbERF-VIII-3a*			n. a.
	Niben101Ctg14204g00003	*NbERF-VIII-3b*	LEP (ERF85)	At5g13910.1	n. a.
	Niben101Scf12639g00001	*NbERF-VIII-4a*	PUCHI (ERF86)	At5g18560.1	n. a.
	Niben101Scf01295g05006	*NbERF-VIII-4b*	ERF87	At1g28160.1	n. a.
	Niben101Scf00941g00007	*NbERF-VIII-5a*	ERF88	At1g12890.1	n. a.
	Niben101Scf19091g00016	*NbERF-VIII-5b*	DRNL (ERF90)	At1g24590.1	n. a.
	Niben101Scf03045g14001	*NbERF-VIII-6*			13
	Niben101Scf02720g08001	*NbERF-VIII-7a*			8
	Niben101Scf08050g00012	*NbERF-VIII-7b*			3
	Niben101Scf08597g01023	*NbERF-VIII-8a*			13
	Niben101Scf03657g02001	*NbERF-VIII-8b*			n. a.
	Niben101Scf02543g00002	*NbERF-VIII-9*			8
	Niben101Scf12483g01009	*NbERF-VIII-10a*			13
	Niben101Scf01767g03004	*NbERF-VIII-10b*	ERF3 (ERF82)	At1g50640.1	13
	Niben101Scf07310g01001	*NbERF-VIII-11*	ERF7 (ERF83)	At3g20310.1	3
	Niben101Scf05692g06003	*NbERF-VIII-12a*			3
	Niben101Scf02073g01009	*NbERF-VIII-12b*			n. a.
	Niben101Scf09236g00001	*NbERF-VIII-13a*			8
	Niben101Scf01752g05003	*NbERF-VIII-13b*			3
	Niben101Scf06200g01006	*NbERF-VIII-14a*			n. a.
	Niben101Scf19266g01015	*NbERF-VIII-14b*			n. a.
	Niben101Scf06200g00001	*NbERF-VIII-15a*			**2**
	Niben101Scf19266g00006	*NbERF-VIII-15b*			n. a.
	Niben101Scf13779g00003	*NbERF-VIII-16a*	ERF11 (ERF76)	At1g28370.1	13
	Niben101Scf07896g01006	*NbERF-VIII-16b*	ERF10 (ERF77)	At1g03800.1	3
	Niben101Scf09704g02001	*NbERF-VIII-17a*	ERF4/RAP2.5 (ERF78)	At3g15210.1	**16**
	Niben101Scf04350g04004	*NbERF-VIII-17b*	ERF8 (ERF79)	At1g53170.1	3
	Niben101Scf11424g01001	*NbERF-VIII-18a*	ERF9 (ERF80)	At5g44210.1	3
	Niben101Scf09191g00005	*NbERF-VIII-18b*	ERF12 (ERF81)	At1g28360.1	3
	Niben101Scf01426g00001	*NbERF-VIII-19a*			**4**
	Niben101Scf03035g01006	*NbERF-VIII-19b*			**2**
	Niben101Scf05480g00003	*NbERF-VIII-20a*			**4**
	Niben101Scf01008g00004	*NbERF-VIII-20b*			**4**
ERF-IX	Niben101Scf07761g00004	*NbERF-IX-1a*			n. a.
	Niben101Scf01956g12014	*NbERF-IX-1b*	-	-	n. a.
	Niben101Scf01177g04007	*NbERF-IX-2*			13
	Niben101Scf08965g00003	*NbERF-IX-3a*			3
	Niben101Scf06413g01002	*NbERF-IX-3b*			n. a.
	Niben101Scf36372g00002	*NbERF-IX-4a*			7
	Niben101Scf06413g01001	*NbERF-IX-4b*			n. a.
	Niben101Scf01947g01005	*NbERF-IX-5a*			19
	Niben101Scf13455g01002	*NbERF-IX-5b*			15
	Niben101Scf01947g01008	*NbERF-IX-6*	ERF1 (ERF92)	At3g23240.1	**4**
	Niben101Scf15451g00001	*NbERF-IX-7*	ERF15 (ERF93)	At2g31230.1	**14**
	Niben101Scf08141g01011	*NbERF-IX-8a*	ORA59 (ERF94)	At1g06160.1	**10**
	Niben101Ctg04506g00002	*NbERF-IX-8b*			**2**
	Niben101Scf01795g06002	*NbERF-IX-9a*			n. a.
	Niben101Scf06328g00004	*NbERF-IX-9b*			n. a.
	Niben101Scf02063g05001	*NbERF-IX-10a*			**4**
	Niben101Scf07761g02006	*NbERF-IX-10b*			**4**
	Niben101Scf01090g02001	*NbERF-IX-11a*			n. a.
	Niben101Scf17204g01001	*NbERF-IX-11b*			**14**
	Niben101Scf07105g04007	*NbERF-IX-12*	ESE1 (ERF95)	At3g23220.1	n. a.
	Niben101Scf07761g00005	*NbERF-IX-13*	ERF96	At5g43410.1	n. a.
	Niben101Scf01428g04013	*NbERF-IX-14a*	ERF14 (ERF97)	At1g04370.1	n. a.
	Niben101Scf01956g10015	*NbERF-IX-14b*			n. a.
	Niben101Scf17204g01003	NbERF-IX-15a	TDR1 (ERF98)	At3g23230.1	n. a.
	Niben101Scf01090g00001	NbERF-IX-15b			n. a.

	Niben101Scf01212g03005	*NbERF-IX-16a*			**14**
	Niben101Scf01094g03014	*NbERF-IX-16b*			n. a.
	Niben101Ctg16023g00001	*NbERF-IX-17a*			n. a.
	Niben101Scf01956g12018	*NbERF-IX-17b*	ERF91	At4g18450.1	n. a.
	Niben101Scf06413g02012	*NbERF-IX-18*			n. a.
	Niben101Scf01212g03003	*NbERF-IX-19a*			n. a.
	Niben101Scf01094g03016	*NbERF-IX-19b*			n. a.
	Niben101Scf05173g07004	*NbERF-IX-20*			n. a.
	Niben101Scf01159g00002	*NbERF-IX-21a*			n. a.
	Niben101Scf05173g05003	*NbERF-IX-21b*			n. a.
	Niben101Scf05173g07005	*NbERF-IX-22*			n. a.
	Niben101Scf05173g08003	*NbERF-IX-23*			n. a.
	Niben101Scf05173g08002	*NbERF1/NbERF-IX-24* [3]			n. a.
	Niben101Scf01082g07003	*NbERF-IX-25a*			11
	Niben101Scf05173g09004	*NbERF-IX-25b*			n. a.
	Niben101Scf01082g04001	*NbERF-IX-26a*	ERF13 (ERF99)	At2g44840.1	n. a.
	Niben101Scf05855g00002	*NbERF-IX-26b*	ERF1A (ERF100)	At4g17500.1	n. a.
	Niben101Scf06951g00004	*NbERF-IX-27*	AtERF2 (ERF101)	At5g47220.1	11
	Niben101Scf19057g00005	*NbERF-IX-28*			n. a.
	Niben101Scf00428g10006	*NbERF-IX-29a*			**14**
	Niben101Scf02775g00003	*NbERF-IX-29b*			n. a.
	Niben101Scf02775g00001	*NbERF-IX-30*			20
	Niben101Scf00428g09009	*NbERF-IX-31a*			3
	Niben101Scf02775g00002	*NbERF-IX-31b*			**16**
	Niben101Scf08546g00001	*NbERF-IX-32*			n. a.
	Niben101Scf00454g04003	*NbERF-IX-33a*			**4**
	Niben101Scf06865g00004	*NbERF-IX-33b*			**16**
	Niben101Scf01177g04031	*NbERF-IX-34*			3
	Niben101Scf00454g03001	*NbERF-IX-35a*			**16**
	Niben101Scf12210g07001	*NbERF-IX-35b*			**4**
	Niben101Scf00163g22003	*NbERF-IX-36*			n. a.
	Niben101Scf00163g22004	*NbERF-IX-37*	AtERF5 (ERF102)	At5g47230.1	**4**
	Niben101Scf08546g05004	*NbERF-IX-38a*	AtERF6 (ERF103)	At4g17490.1	**16**
	Niben101Scf08546g05002	*NbERF-IX-38b*	ERF104	At5g61600.1	**16**
	Niben101Scf00163g22002	*NbERF-IX-39a*	ERF105	At5g51190.1	**16**
	Niben101Scf08546g03002	*NbERF-IX-39b*			**4**
	Niben101Scf23241g00003	*NbERF-IX-40a*			**2**
	Niben101Scf03310g00003	*NbERF-IX-40b*			**4**
	Niben101Scf08546g05005	*NbERF-IX-41*			**4**
	Niben101Scf08249g02004	*NbERF-IX-42*	ERF106	At5g07580.1	7
	Niben101Scf00454g02008	*NbERF-IX-43a*	DEWAX (ERF107)	At5g61590.1	1
	Niben101Scf00454g02007	*NbERF-IX-43b*			6
ERF-X	Niben101Scf04634g00008	*NbERF-X-1a*			6
	Niben101Ctg15010g00002	*NbERF-X-1b*			6
	Niben101Scf01453g11005	*NbERF-X-2a*			18
	Niben101Scf01942g08004	*NbERF-X-2b*			18
	Niben101Scf12210g08011	*NbERF-X-3a*	RAP2.6 (ERF108)	At1g43160.1	**16**
	Niben101Scf02242g03012	*NbERF-X-3b*	ERF110	At5g50080.1	20
	Niben101Scf10438g00010	*NbERF-X-4a*	ABR1 (ERF111)	At5g64750.1	n. a.
	Niben101Scf10438g00009	*NbERF-X-4b*	RAP2.6L (ERF113)	At5g13330.1	20
	Niben101Scf11706g00010	*NbERF-X-5*	ERF114	At5g61890.1	20
	Niben101Scf06621g02032	*NbERF-X-6a*	ERF115	At5g07310.1	9
	Niben101Scf03548g02037	*NbERF-X-6b*			9
	Niben101Scf07579g01012	*NbERF-X-7*			**2**
	Niben101Scf14233g00008	*NbERF-X-8a*			**16**
	Niben101Scf00725g06004	*NbERF-X-8b*			n. a.
	Niben101Scf00321g03008	*NbERF-X-9a*			n. a.
	Niben101Scf02483g02007	*NbERF-X-9b*			n. a.
	Niben101Scf06203g05020	*NbERF-X-10*	RRTF1 (ERF109)	At4g34410.1	n. a.
	Niben101Scf02408g00021	*NbERF-X-11a*	ERF120	At2g20350.1	n. a.
	Niben101Scf07172g00019	*NbERF-X-11b*	ERF121	At5g67010.1	18
	Niben101Scf05140g01007	*NbERF-X-12a*	ERF122	At5g67000.1	n. a.
	Niben101Scf01350g07008	*NbERF-X-12b*			n. a.
	Niben101Scf00839g00006	*NbERF-X-13*			n. a.
Soloist II	Niben101Scf08516g01005	*NbERF-S-1*	ERF84	At1g80580.1	n. a.
Soloist III (DREB-A3)	Niben101Scf05357g00008	*NbERF-S-2a*	ABI4 (ERF52)	At2g40220.1	n. a.
	Niben101Scf06984g02002	*NbERF-S-2b*			n. a.

[1] Cluster numbers for INF1-induced genes are shown in bold.

[2] NbERF173/NbERF-VII-2 was reported as a gene involved in the resistace of *N. benthamiana* against *Phytophthora parasitica* (Yu et al., 2020).

[3] NbERF1/NbERF-IX-24 was reported as a gene involved in the MeJA-induced nicotine biosynthesis (Todd et al., 2010).

n. a., not assigned.
